# Could vector-derived cadherin mimicry contribute to pemphigus vulgaris? An immunogenetic and *in silico* study involving HLA-DRB104:02 and 14:01

**DOI:** 10.3389/fimmu.2026.1745207

**Published:** 2026-02-18

**Authors:** Bayram Toraman, Burak Kaan Kasap, Hande Ermis, Deniz Aksu Arica, Savaş Yayli

**Affiliations:** 1 Faculty of Medicine, Department of Medical Biology, Karadeniz Technical University, Trabzon, Türkiye; 2 Department of Medical Biology, Institute of Health Sciences, Karadeniz Technical University, Trabzon, Türkiye; 3 Department of Dermatology and Venereology, School of Medicine, Koc University, Istanbul, Türkiye; 4 Department of Dermatology and Venereology, Faculty of Medicine, Karadeniz Technical University, Trabzon, Türkiye

**Keywords:** auto antibody, autoimmunity, HLA-DRB1, molecular mimicry, pemphigus vulgaris

## Abstract

**Introduction:**

Pemphigus vulgaris (PV) is a potentially life-threatening autoimmune blistering disease characterized by autoantibodies directed against the desmosomal cadherins desmoglein 3 (DSG3) and desmoglein 1 (DSG1), which are essential for keratinocyte adhesion. While the pathogenic role of these autoantibodies is well established, the upstream events leading to loss of immune tolerance against desmogleins remain incompletely understood. Genetic susceptibility conferred by HLA class II alleles and environmental exposures are thought to interact during disease initiation. In this study, we investigated *HLA-DRB1* allele and genotype associations in Turkish PV patients and explored a hypothesis-generating framework linking genetic susceptibility with environmental exposure. Specifically, we examined whether vector-derived cadherin-like proteins could represent potential molecular mimics of desmogleins in genetically predisposed individuals.

**Methods:**

*HLA-DRB1* allele frequencies were analyzed in 86 PV patients and 200 healthy controls using PCR-SSOP and Luminex-based genotyping. *In silico* analyses included MHC class II peptide-binding prediction (IEDB), structural modeling using AlphaFold, and molecular dynamics simulations performed with GROMACS. These computational approaches were applied to evaluate structural similarity and relative binding compatibility between cadherin-like peptides derived from hematophagous vectors and disease-associated *HLA-DRB1* alleles, including *04:02 and *14:01.

**Results:**

HLA-DRB1*04:02 and HLA-DRB1*14:01 alleles were significantly enriched in PV patients compared with controls, and the heterozygous HLA-DRB1*04:02/14:01 genotype was overrepresented in the patient cohort. Structural and energetic analyses indicated that selected vector-derived cadherin-like peptides can adopt stable conformations when bound to these HLA variants and display conformational features compatible with DSG3 ectodomain–derived peptides.

**Discussion:**

These findings highlight strong immunogenetic associations in PV and introduce a structurally and immunogenetically informed, hypothesis-generating Vector-derived Cadherin Mimicry (VCM) framework. By integrating genetic association data with computational modeling, this conceptual model suggests that repeated exposure to vector-derived cadherin-like proteins may represent a plausible environmental component contributing to PV susceptibility in genetically predisposed individuals. Experimental validation will be required to further evaluate this framework and its relevance to PV pathogenesis.

## 1 Introduction

Autoimmune diseases emerge when self-tolerance is lost due to interactions between genetic susceptibility and environmental triggers. Pemphigus vulgaris (PV) exemplifies organ-specific autoimmunity, characterized by pathogenic autoantibodies against desmoglein 3 (DSG3) and desmoglein 1 (DSG1), desmosomal cadherins vital for epithelial adhesion (1, 2). Binding of these antibodies disrupts keratinocyte junctions, causing acantholysis and intraepidermal blistering (3, 4). Although autoantibody pathogenicity is well defined, the mechanisms leading to loss of tolerance remain poorly understood (5). In PV, defects in both central and peripheral tolerance mechanisms are believed to contribute to disease onset. Central tolerance occurs in the thymus for T cells and in the bone marrow for B cells (6, 7). Defects in central and peripheral tolerance may contribute to PV onset. Incomplete deletion of autoreactive T cells in the thymus or B cells in the bone marrow may allow self-reactive clones to persist. Failures in peripheral tolerance mechanisms—such as anergy, regulatory T cell (Treg) suppression, or activation-induced cell death—can permit their activation (3–5, 7). Furthermore, epitope spreading, where immune responses expand from one epitope to others within or across proteins, contributes to disease progression. In PV, this is exemplified by the progression of immune responses from DSG3 to DSG1 epitopes, reflecting a clinical shift from mucosal-limited disease to the mucocutaneous form, which often correlates with increased disease severity and resistance to therapy (8, 9).

PV shows a consistent genetic link with HLA-DRB1*04:02 and *14:01 alleles, reported in multiple populations. These alleles likely facilitate presentation of DSG3 peptides to autoreactive CD4+ T cells (10–12). In addition to genetic predisposition, environmental factors are thought to play a contributory role in triggering or perpetuating the autoimmune response. In addition, environmental exposure may act as a secondary trigger. Molecular mimicry—a phenomenon in which microbial or environmental antigens share structural similarity with self-proteins—has been proposed as one mechanism driving autoimmunity (1, 3, 5, 8, 13). Endemic pemphigus foliaceus (fogo selvagem) in Brazil is one such example where sandfly (*Lutzomyia longipalpis*) salivary antigen (LJM11) has been implicated as a potential trigger due to its sequence resemblance to DSG1 (13, 14). Among blood-feeding vectors, exposure to *Simulium nigrimanum* (black fly) bites was shown to correlate with anti-desmoglein titers in an epidemiological study (15). However, no equivalent mimicry mechanism has been demonstrated for PV or DSG3-specific autoimmunity.

Here, we conducted a genetic association study to evaluate *HLA-DRB1* allele frequencies in Turkish PV patients and matched controls. Our findings revealed a strong association between PV and the HLA-DRB1*04:02 and HLA-DRB1*14:01 alleles, consistent with previous reports in other populations. Building on these observations, we advance a hypothesis in which cadherin-like proteins from the salivary glands of mosquitoes or other hematophagous vectors (e.g., sandflies) may share structural features with DSG3/DSG1. In this proposed “vector-derived cadherin mimicry” (VCM) framework, structural similarity—rather than sequence identity—is considered a central element of potential mimicry. Within this context, repeated or chronic antigen exposure in genetically predisposed hosts could be compatible with scenarios leading to loss of tolerance and the emergence of PV-associated autoimmunity, without implying a deterministic or universal mechanism. To explore the structural and immunogenetic plausibility of this framework, we integrated HLA genotyping, peptide-binding predictions, structural modeling, and molecular dynamics analyses.

## 2 Materials and methods

### 2.1 Study population

This case–control study included patients diagnosed with pemphigus vulgaris (PV) at the Bullous Diseases outpatient clinic, Department of Dermatology and Venereology, Karadeniz Technical University (KTU). Diagnosis was confirmed by clinical findings, histopathology, direct immunofluorescence, and/or ELISA. Only patients without other autoimmune diseases who provided informed consent were included. In total, 86 PV patients (aged 21–81 years) and 200 age- and sex-matched healthy controls participated. Peripheral blood samples (5–9 ml) were collected in EDTA tubes for genomic DNA extraction using the MiniFavorPrep™ kit (Favorgen, Australia). The study was approved by the KTU Faculty of Medicine Ethics Committee (protocol no: 2022/51).

### 2.2 HLA-DRB1 genotyping and statistical analysis

*HLA-DRB1* typing was performed by sequence-specific oligonucleotide probe (PCR-SSOP) using the Luminex platform (LABScan3D, One Lambda, USA) and analyzed with HLA Fusion software. The One Lambda SSP^®^ kit (Thermo Fisher Scientific) was used for allele identification (16, 17). Power analysis was conducted using the PGA v2.0 program to ensure 80% statistical power software (18). Hardy–Weinberg equilibrium in controls was tested with GENEPOP (19). Allele and genotype frequencies were compared using Pearson’s chi-square or Fisher’s exact test when expected counts were <5, implemented in RStudio (v2024.04.2). Significance was adjusted with the Benjamini–Hochberg FDR method; p_adj < 0.05 was considered significant.

### 2.3 Identification of cadherin-like homologs and antigenic peptide prediction

To identify candidate cadherin-like proteins with potential for molecular mimicry, we performed BLASTP searches against the VectorBase database (https://vectorbase.org/vectorbase/app) using the EC1 domain of human DSG3 as the query sequence. We employed PSI-BLAST to increase sensitivity for detecting structurally conserved and evolutionarily related sequences. PSI-BLAST utilize position-specific scoring matrices to capture deeper pattern-level homology not evident from linear sequence identity alone. The top-scoring result was a cadherin-like protein from *Aedes albopictus*, annotated based on homology to *Aedes aegypti* (protein: AAEL000597; Gene symbol: LOC5564848/neural-cadherin) (**Supplementary File**). This protein, with a predicted length of 1569 amino acids, was selected for further analysis based on its high similarity score and cadherin domain architecture. From the BLAST alignment, we extracted the homologous region to human DSG3 ectodomain 1 (hDSG3 EC1) and converted it to FASTA format. This sequence was subjected to MHC class II peptide-binding predictions using the IEDB MHC-II binding tool (www.iedb.org), applying the same parameters used for hDSG3. Based on our and other genetic association findings, HLA-DRB1*04:02 and HLA-DRB1*14:01 were selected as the MHC class II alleles of interest. Candidate antigenic peptides were prioritized by (1): strong predicted binding affinity (2), high immunogenicity scores, and (3) maximal alignment similarity with hDSG3 EC1 sequences (20, 21).

### 2.4 Structural modeling, molecular dynamics and binding free energy calculations

Structural modeling of EC1 peptides from hDSG3 and *Aedes albopictus* cadherin-like protein was performed using AlphaFold3 and AlphaFoldMultimer tools (22, 23) on the Colab platform (24). Molecular models were visualized using UCSF ChimeraX (v1.10) (25). All-atom molecular dynamics (MD) simulations were carried out using GROMACS (v2022.5) with the AMBER99SB-ILDN force field. Systems were solvated with TIP3P water, neutralized, energy-minimized, equilibrated, and subjected to a 100 ns production run at 298 K and 1 atm using the V-rescale thermostat and Parrinello–Rahman barostat (26). MD trajectories were analyzed to characterize structural stability and residue-level flexibility of the peptide–HLA complexes. Peptide backbone root mean square deviation (RMSD) was calculated following alignment of each trajectory frame to the HLA backbone atoms. Root mean square fluctuation (RMSF) was computed for peptide residues using the equilibrated portion of the trajectories. For all analyses, the final 47.5 ns of the 100 ns production simulations were considered. Detailed trajectory analysis procedures, including atom selection criteria and alignment settings, are provided in the **Supplementary Methods**.

Binding free energies (ΔG_binding) for peptide–protein complexes were estimated using gmx_MMPBSA (v1.5.7) with the MM/GBSA approach (GB-OBC II, igb=5). Representative frames from the equilibrated trajectories were used for binding energy estimation and per-residue energy decomposition analyses (27, 28). All additional parameters, scripts, and analysis settings are detailed in the **Supplementary File**.

## 3 Results

### 3.1 HLA-DRB1 alleles and genotype statistics

In the control group, *HLA-DRB1* alleles were in Hardy–Weinberg equilibrium. A total of 33 distinct *HLA-DRB1* alleles were detected across all participants (**Table 1**). The ten most frequent alleles in PV patients and controls are listed in **Table 1**. The most common alleles observed in PV patients were HLA-DRB1*04:02 (40%) and HLA-DRB1*14:01 (18%). In controls, the predominant alleles were HLA-DRB1*11:01, 11:04, and 07:01 (each approximately 9%). Allele frequencies and corresponding statistical analyses are summarized in **Table 2**. Carriage of HLA-DRB1*04:02 was significantly associated with PV in this cohort (OR = 12.95; 95% CI: 7.40–24.57). Similarly, HLA-DRB1*14:01 was more frequent among PV patients compared with controls (p = 1.23×10^-5^; OR = 4.55; 95% CI: 2.35–8.74). Genotype analysis was subsequently performed to evaluate the co-occurrence of both alleles (HLA-DRB1*04:02/14:01). Genotype frequencies and statistical results are shown in **Table 3**. The heterozygous HLA-DRB1*04:02/14:01 genotype was observed in PV patients but not detected among controls. This distribution reflects an enrichment of this genotype in the patient group within the studied cohort; however, estimates based on low genotype counts should be interpreted with caution.

**Table 1:** The top 10 most frequently observed *HLA-DRB1* alleles in PV patients and controls.

HLA-DRB1*	PV (n)	%	HLA-DRB1*	Control (n)	%
**04:02**	70	40.70	11:01	39	9
**14:01**	31	18.02	11:04	39	9
11:04	9	5	07:01	37	9
01:01	7	4	15:01	33	8
04:03	7	4	03:01	26	6
07:01	7	4	16:01	26	6
13:02	7	4	01:01	23	5
11:01	6	3	13:01	19	4
15:01	6	3	**04:02**	18	4.50
03:01	5	2	**14:01**	17	4.25
**Total**	**155**	**87.72**		**277**	**64.75**

n, number of alleles.

Bold values means statistically significant findings.

**Table 2:** HLA-DRB1 allele frequencies and chi-square test results.

HLA-DRB1*	Control (n)	PV (n)	p	adj_p	OR	95% CI
**04:02**	**18**	**70**	**2.03×10^-24^**	**6.71×10^-^²³**	**12.95**	**7.40-24.57**
**14:01**	**17**	**31**	**7.51×10^-7^**	**1.23×10^-5^**	**4.55**	**2.35-8.74**
**16:01**	**26**	**0**	**9.75×10^-5^**	**0.001**	**0.03**	**0-0.31**
**11:01**	**39**	**6**	**0.00**	**0.037**	**0.33**	**0.10-0.75**
14:04	0	4	0.00	0.06	20.19	1.46-∞
15:01	33	6	0.02	0.10	0.40	0.12-0.91
13:03	12	0	0.02	0.10	0.08	0-0.77
07:01	37	7	0.03	0.13	0.41	0.17-0.97
09:01	8	0	0.06	0.22	0.12	0-1.27
04:01	16	1	0.07	0.23	0.19	0.029-1.14
11:04	39	9	0.07	0.23	0.50	0.22-1.09
08:01	0	1	0.09	0.25	6.61	0.40-∞
11:11	0	1	0.09	0.25	6.61	0.40-∞
03:01	26	5	0.12	0.28	0.43	0.16-1.23
04:05	6	0	0.18	0.37	0.16	0-1.86
04:08	5	0	0.18	0.37	0.19	0-1.86
15:02	12	1	0.24	0.37	0.25	0.03-1.62
10:01	11	2	0.24	0.44	0.46	0.03-1.64
04:04	4	4	0.26	0.46	2.22	0.40-12.06
04:03	9	7	0.30	0.49	1.76	0.59-5.10
04:07	3	0	0.31	0.49	0.31	0-3.34
13:02	12	7	0.46	0.69	1.3	0.51-3.98
13:01	19	6	0.51	0.74	0.72	0.21-1.72
01:01	23	7	0.55	0.76	0.68	0.27-1.67
08:04	2	1	0.59	0.78	1.31	0.15-30.33
01:02	3	2	1	1	1.57	0.1-7.80
04:06	1	0	1	1	0.72	0-11.78
13:05	1	0	1	1	0.72	0-11.78
16:02	1	0	1	1	0.72	0-11.78
08:02	2	0	1	1	0.43	0-11.70
08:03	7	3	1	1	1.02	0.24-4.15
11:03	6	1	1	1	0.50	0.07-4.01
12:01	2	0	1	1	0.43	0-11.71

Bold values means statistically significant findings.

**Table 3:** The most frequently observed genotypes and Fisher’s exact test statistics in PV patients and controls in the study.

DRB1 Genotype	Control (n)	PV (n)	a*	b*	c*	d*	p	adj_p	OR	%95 CI
**04:02/14:01**	**0**	**20**	**20.5**	**0.5**	**71.5**	**200.5**	**1.63 × 10^-^¹¹**	**2.21 × 10^-9^**	**114.97**	**6.86 – 1925.82**
**04:02/04:03**	**0**	**6**	**6.5**	**0.5**	**85.5**	**200.5**	**0.0008**	**0.0374**	**30.48**	**1.69 – 547.22**
**04:02/13:02**	**0**	**6**	**6.5**	**0.5**	**85.5**	**200.5**	**0.0008**	**0.0374**	**30.48**	**1.69 – 547.22**
01:01/04:02	0	5	5.5	0.5	86.5	200.5	0.0027	0.0934	25.49	1.39 – 466.2
04:02/04:04	0	3	3.5	0.5	88.5	200.5	0.0298	0.6670	15.85	0.81 – 310.28
04:02/13:01	0	4	4.5	0.5	87.5	200.5	0.0091	0.2465	20.62	1.09 – 387.21
11:04/11:04	12	1	1.5	12.5	90.5	188.5	0.0700	1	0.24	0.04 – 1.38
15:01/15:01	7	0	0.5	7.5	91.5	193.5	0.1027	1	0.14	0.007 – 2.49
07:01/11:01	5	0	0.5	5.5	91.5	195.5	0.3294	1	0.19	0.01 – 3.55

*0.5 was added to the cell frequencies as part of the Haldane-Anscombe correction. Therefore, the upper limit of the confidence interval was calculated as infinity. n, number of genotype; adj_p, corrected p-value; OR, Risk ratio; CI, confidence interval.

Bold values means statistically significant findings.

### 3.2 hDSG3 EC1 and *Aedes albopictus* EC1-like domain antigenic peptide prediction

**Figure 1:**
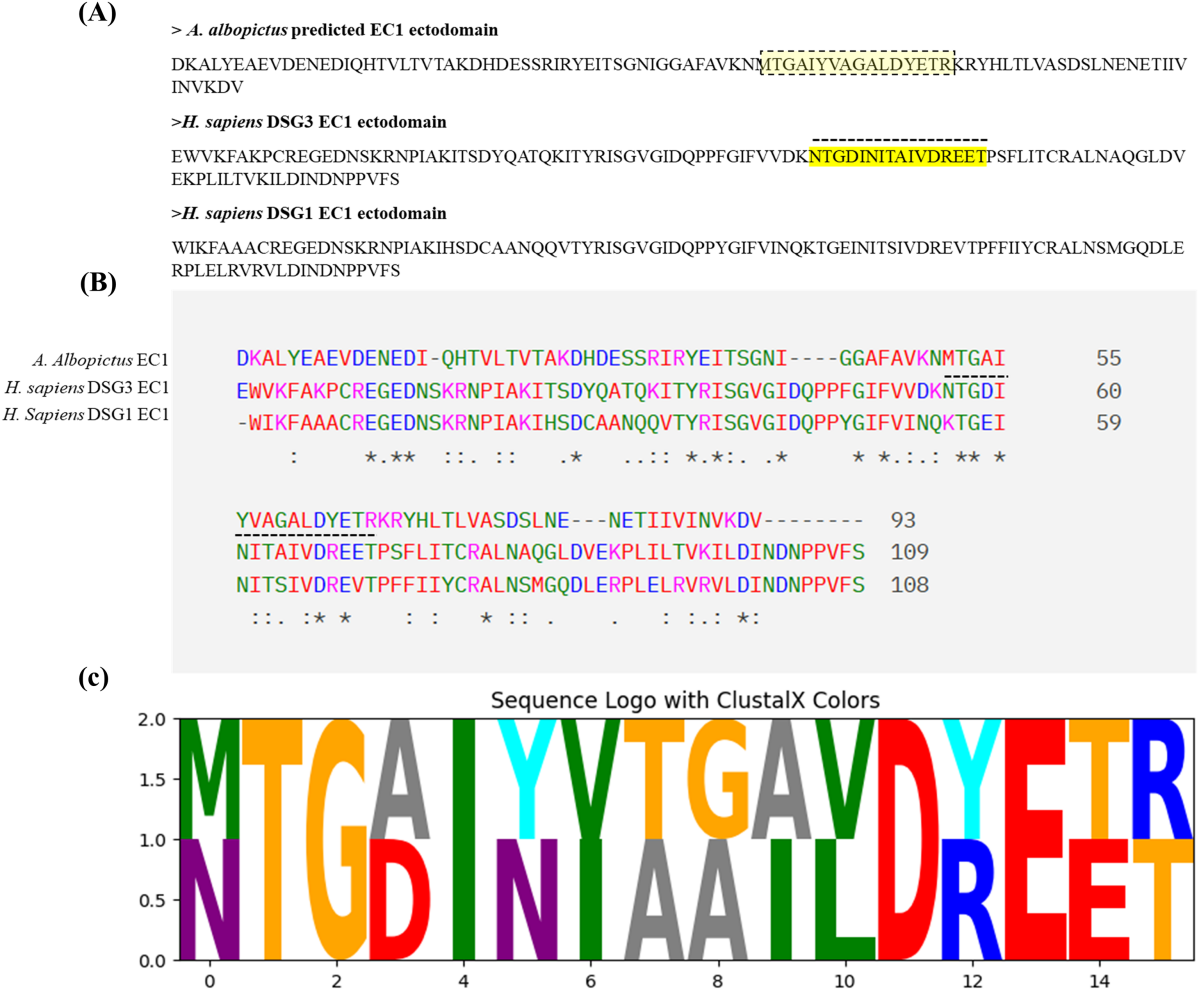
Comparative analysis of the EC1 domains of hDSG3, hDSG1 and **(A)***albopictus* cadherin-like protein reveals a conserved antigenic motif. **(A)** Schematic representation of the EC1 domain of hDSG3, hDSG1 and **(A)***albopictus*, showing the most antigenic 16-mer peptides identified using the IEDB MHC-II binding prediction tool (highlighted in yellow). **(B)** Multiple sequence alignment of hDSG3, hDSG1 and EC1 domains with the **(A)***albopictus* EC1-like domain. The highlighted regions correspond to the predicted peptides shown in panel **(A)** Alignment was performed using Clustal Omega, and conserved residues are indicated below the sequences. **(C)** Sequence logo comparing the selected peptides from Aa_P (MTGAIYVAGALDYETR) and hDSG3 (NTGDINITAIVDREET). The height of each letter reflects the relative frequency and conservation of the residue at that position. Coloring follows the ClustalX scheme, with residues colored based on their physicochemical properties: hydrophobic (green), small/hydroxyl (orange), acidic (red), basic (blue), polar (cyan), aromatic (magenta), and others (gray)—sequence logo generated using the Python-based Logomaker library (v0.8) in Google Colab.

MHC class II peptide-binding predictions performed using the IEDB tool identified 19 unique predicted peptides within the hDSG3 EC1 domain and 167 within the *A. albopictus* EC1-like domain (Aa_P) (**Supplementary Table S1**). From these predicted peptides, candidates were prioritized based on a combination of predicted binding affinity, immunogenicity score, and sequence similarity to the hDSG3 EC1 domain (**Figure 1**).

### 3.3 Structural modeling and binding free energy analysis

**Figure 2:**
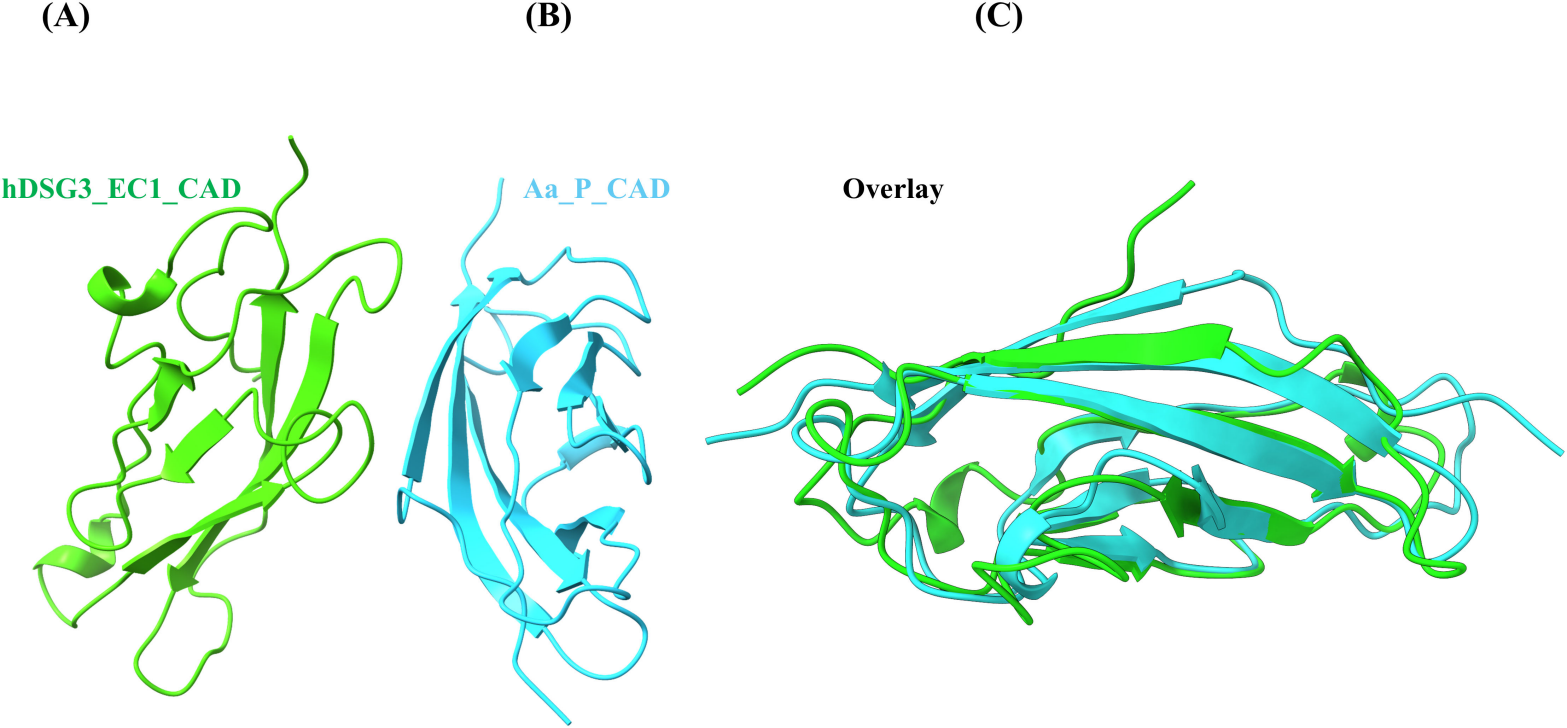
Structural comparison and alignment of EC1 cadherin domains from hDSG3 and Aedes albopictus. **(A)** Ribbon representation of the extracellular cadherin 1 (EC1) domain from human desmoglein-3 (hDSG3_EC1_CAD), predicted using AlphaFold. **(B)** Ribbon representation of the EC1-like domain from Aedes albopictus cadherin-like protein (Aa_P_CAD), based on AlphaFold modeling and sequence homology to the human DSG3 EC1 domain. **(C)** Structural overlay of the two domains, highlighting conserved β-strand architecture and overall topological similarity. Overlay was generated by pairwise structural alignment using UCSF ChimeraX (v1.10), employing default Needleman–Wunsch alignment and backbone RMSD minimization. Color scheme: hDSG3 (green), Aa_P (cyan). The high degree of β-sheet preservation supports the hypothesis that vector-derived cadherin-like domains may mimic key conformational features of desmogleins, potentially enabling molecular mimicry in the context of HLA-mediated antigen presentation.

**Figure 3:**
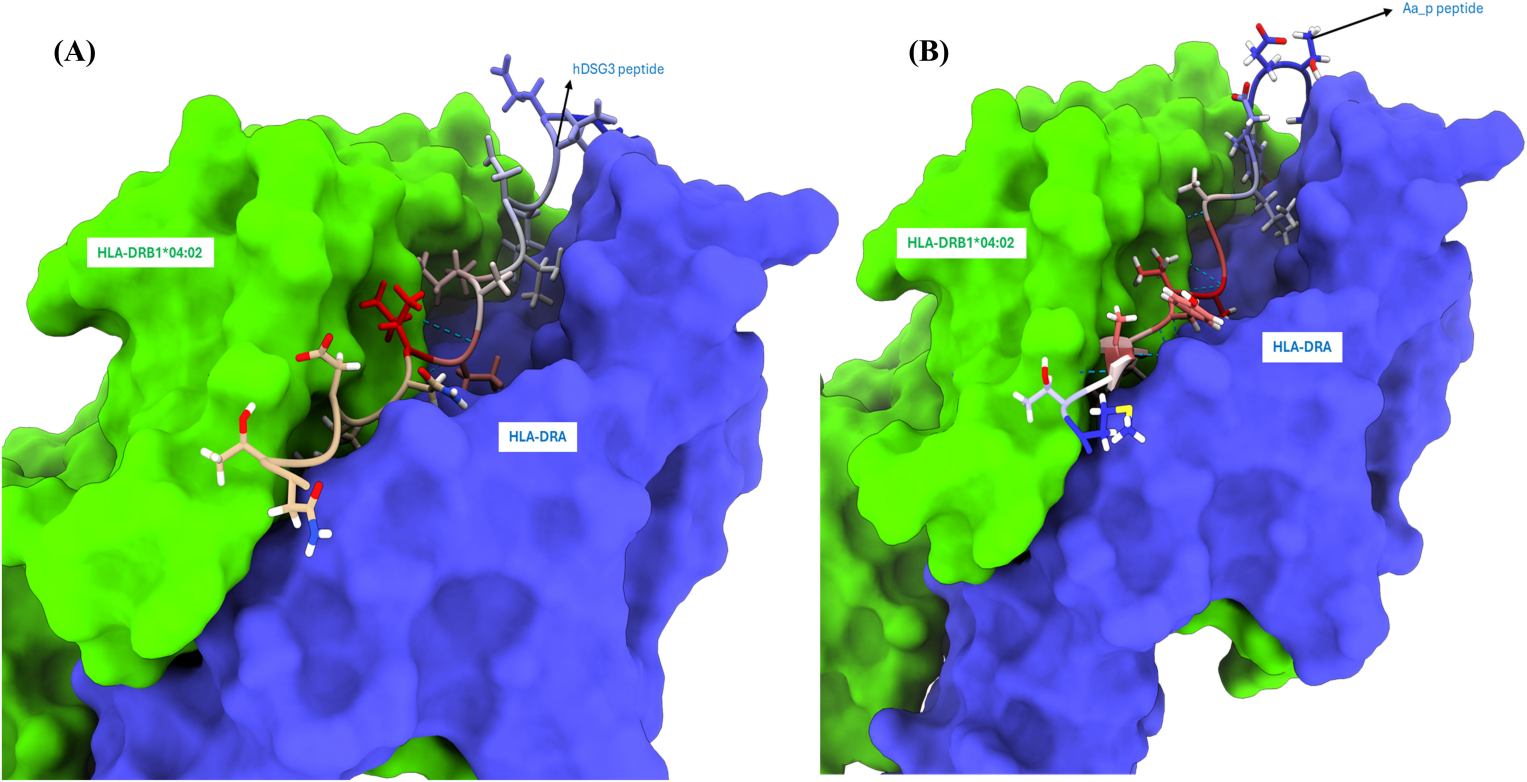
Surface representation of the HLA-DR heterodimers **(A)** Molecular surface rendering of the HLA-DR heterodimer complex comprising the α-chain (HLA-DRA, blue) and β-chain (HLA-DRB1*04:02, green) bound to a representative peptide derived from the EC1 domain of human desmoglein 3 (hDSG3). The peptide is shown as a stick model within the peptide-binding groove. **(B)** Equivalent surface rendering of the same HLA-DR heterodimer bound to a predicted immunogenic peptide from the EC1-like domain of a cadherin-like protein in *Aedes albopictus* (Aa_P peptide). The vector-derived peptide adopts a similar binding pose within the groove, demonstrating structural compatibility. Models were generated using AlphaFold-Multimer and visualized with UCSF ChimeraX (v1.10). Structural alignments and docking were manually refined to preserve canonical MHC class II geometry. Conserved binding orientation and similar residue positioning between peptides support the hypothesis of structural mimicry between vector-derived and self-antigens, relevant to HLA-DRB1*04:02–restricted immune responses.

Structural comparison of the modeled hDSG3 and *A. albopictus* EC1 domains revealed a high degree of similarity in overall domain architecture and secondary structure (**Figures 2A-C**; **Supplementary File**). Following docking of the predicted peptides to HLA-DRB1*04:02, stable receptor–ligand complexes were obtained for both peptide–HLA pairs (**Figure 3**). Subsequent molecular dynamics simulations indicated comparable binding poses for the two peptides throughout the analyzed trajectories. Despite these structural similarities, calculated binding free energies differed between the two complexes (**Table 4**).

**Table 4:** Binding free energies of both complexes (kcal/mol).

Component	Mean_ hDSG3	Mean_ Aa_P	Difference (Aa_P - hDSG3)
VDWAALS	-66.79	-109.68	-42.89
EEL	38.06	36.93	-1.13
EGB	3.54	-0.03	-3.57
ESURF	-9.85	-14.72	-4.87
GGAS	-28.73	-72.74	-44.01
GSOLV	-6.31	-14.75	-8.44
**TOTAL**	**-35.04**	**-87.5**	**-52.46 kcal/mol**

VDWAALS, Van der Waals; EEL, Electrostatic; EGB, GB solvent effect.

ESURF, Solvent surface energy; GGAS, Internal energy (VDW + EEL).

GSOLV, Solvation energy (EGB + ESURF); TOTAL, Bonding ΔG.

Bold values means statistically significant findings.

### 3.4 Molecular dynamics stability and flexibility analyses

**Figure 4:**
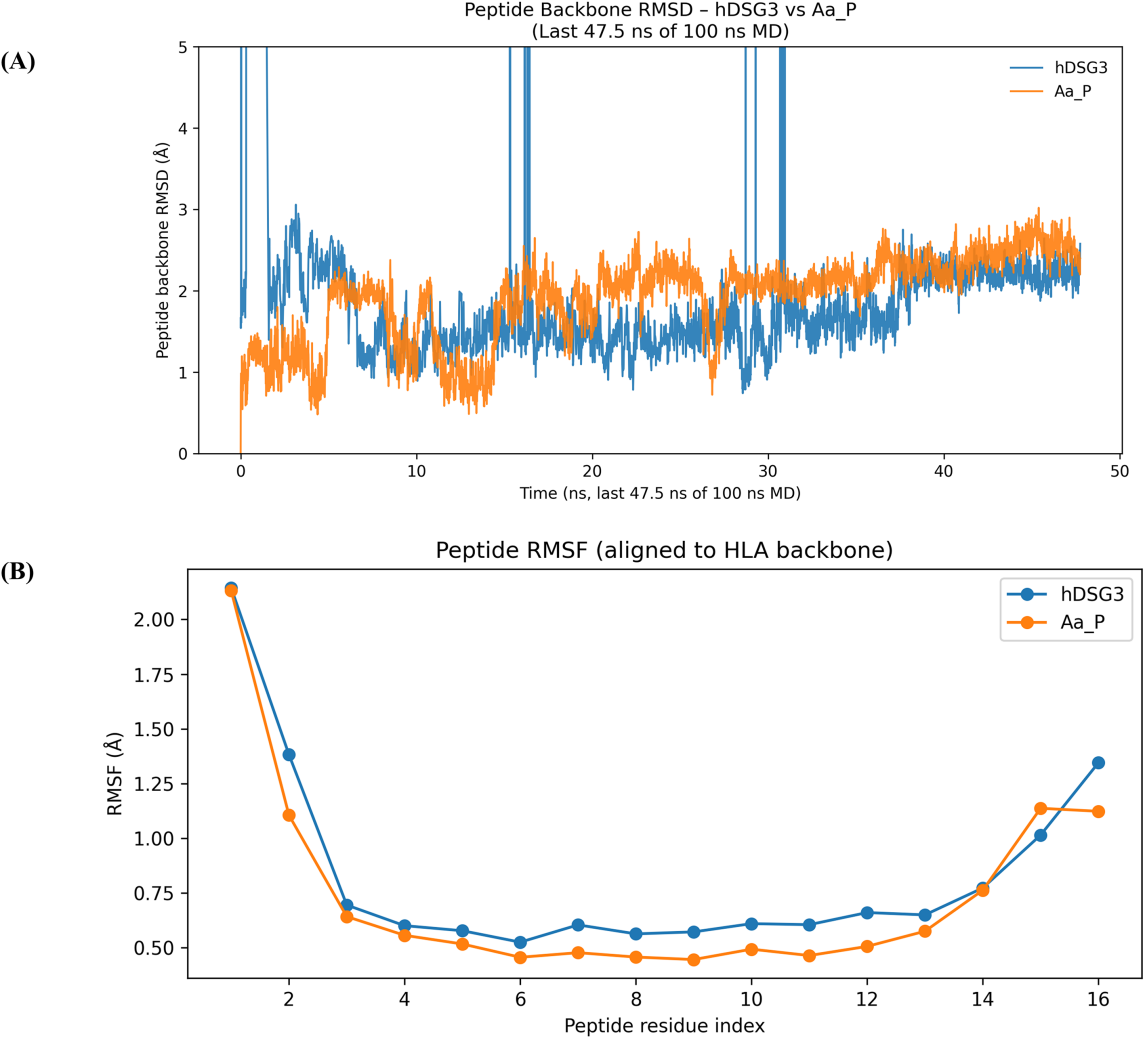
Molecular dynamics stability and flexibility analyses of peptide–HLA complexes. **(A)** Peptide backbone RMSD profiles for the hDSG3 EC1-derived peptide and the Aa_P peptide, calculated over the equilibrated phase of the MD simulations (last 47.5 ns of 100 ns), following alignment to the HLA backbone. Both peptides exhibit comparable RMSD ranges without evidence of sustained structural drift. **(B)** Peptide RMSF profiles calculated over the equilibrated portion of the trajectories. Both peptides show low to moderate residue-level fluctuations within the HLA binding groove, with increased flexibility primarily confined to terminal residues.

The dynamic stability of the peptide–HLA complexes was evaluated by peptide backbone root mean square deviation (RMSD) analysis following alignment to the HLA backbone (**Figure 4A**). RMSD traces calculated over the equilibrated portion of the trajectories (last 47.5 ns) indicated that both the hDSG3 EC1-derived peptide and the Aa_P peptide fluctuated within comparable RMSD ranges, without evidence of sustained structural drift. Occasional transient RMSD excursions were observed for the hDSG3 EC1-derived peptide during the analyzed window; however, these fluctuations were short-lived and did not result in a sustained increase in RMSD, supporting comparable backbone stability of both peptides under the simulated conditions. Residue-level flexibility of the bound peptides was assessed by root mean square fluctuation (RMSF) analysis after alignment to the HLA backbone (**Figure 4B**). Both the Aa_P and hDSG3 EC1-derived peptides exhibited low RMSF values across the central region of the peptide sequence, indicating constrained fluctuations within the HLA binding groove. Increased flexibility was primarily observed at the N- and C-terminal residues for both peptides, a pattern commonly associated with reduced anchoring at peptide termini. Overall, the RMSF profiles of the two peptides were broadly similar, with only modest residue-specific differences. No pronounced or regionally distinct increases in flexibility were observed for either peptide, suggesting comparable dynamic accommodation within the HLA binding site under the simulated conditions.

### 3.5 *In silico* evaluation of the VCM hypothesis

**Figure 5:**
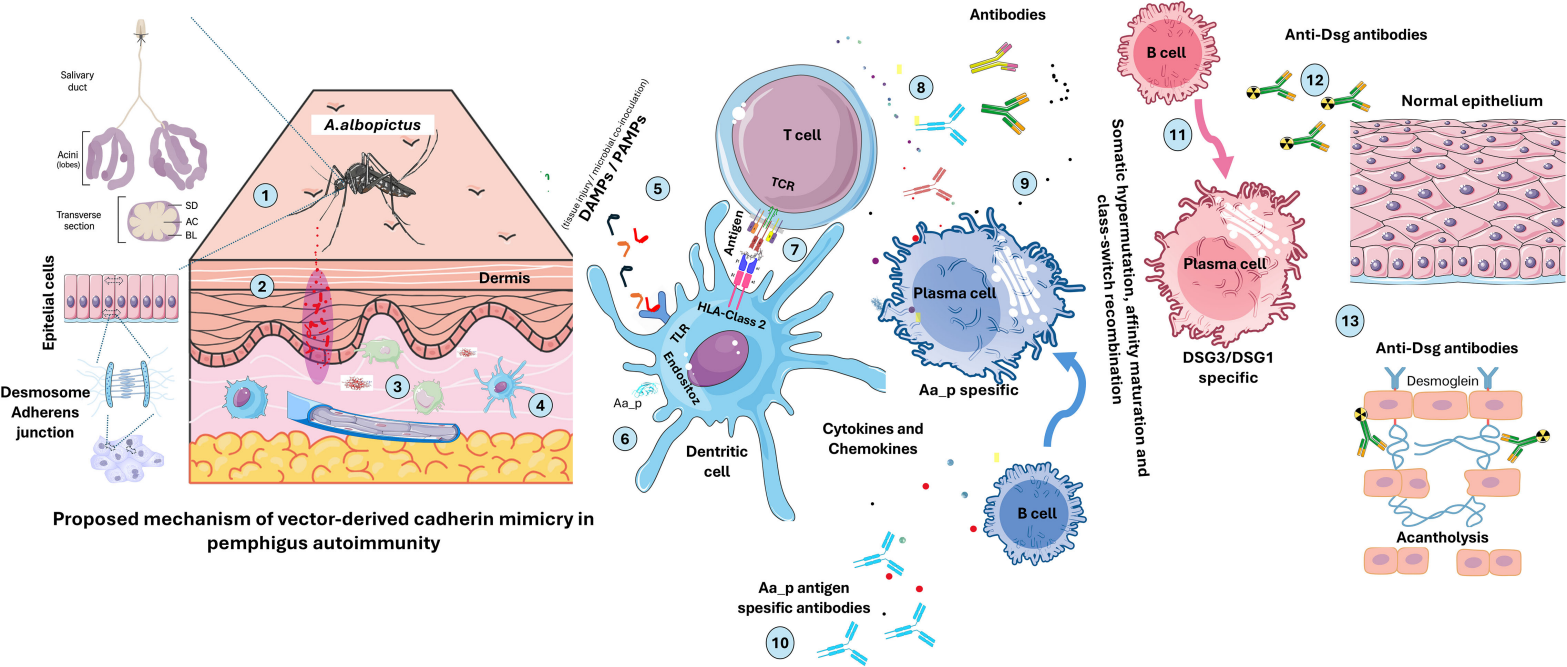
Proposed model of Vector-derived Cadherin Mimicry (VCM) in pemphigus vulgaris. This schematic illustrates a hypothesis-generating, non-deterministic model describing how exposure to hematophagous vector–derived cadherin-like proteins could contribute to PV in genetically susceptible individuals. It is intended to illustrate a conceptual framework supported by *in silico* analyses and existing transcriptomic observations, highlighting testable hypotheses for future experimental investigation. 1. *Aedes albopictus* introduces salivary components into the host skin during blood feeding. 2. Vector saliva, together with tissue injury at the bite site, leads to deposition of salivary molecules and potential cadherin-like peptide candidates within the dermis. 3. Local tissue perturbation and cellular stress may result in the release of damage-associated molecular patterns (DAMPs) and, in some contexts, pathogen-associated molecular patterns (PAMPs) derived from microbial co-inoculation. 4. Resident antigen-presenting cells (APCs), including dendritic cells, encounter vector-derived peptides in an inflammatory microenvironment shaped by tissue damage and innate immune cues. 5. Innate immune sensing occurs through pattern-recognition receptors (e.g., Toll-like receptors), responding to DAMPs/PAMPs, promoting APC activation and cytokine production. 6. Vector-derived cadherin-like peptides (Aa_P) are internalized by APCs and processed via the endosomal pathway. 7. Processed peptides are presented as peptide–HLA class II complexes, including complexes involving PV-associated alleles such as HLA-DRB1*04:02 and HLA-DRB1*14:01, and recognized by CD4^+^ T cells through the T-cell receptor (TCR). 8. Activated CD4^+^ T cells provide cytokines and co-stimulatory signals that support B-cell activation. 9. B cells recognizing vector-derived cadherin-like peptides undergo clonal expansion and differentiation, including germinal-center processes such as somatic hypermutation and class-switch recombination. 10. Plasma cells initially produce antibodies directed against vector-derived antigens. 11. Through molecular mimicry and affinity maturation, a subset of antibodies may acquire cross-reactivity toward desmoglein 3 (DSG3) and/or desmoglein 1 (DSG1). 12. Anti-desmoglein autoantibodies bind to desmosomal cadherins on keratinocyte surfaces in the epidermis. 13. Autoantibody binding interferes with desmoglein-mediated cell–cell adhesion, contributing to acantholysis and intraepidermal blister formation characteristic of PV.

To assess the plausibility of the VCM hypothesis, we constructed HLA-DRA/DRB1*04:02–peptide complexes and performed peptide-binding predictions, structural modeling, and molecular dynamics simulations. Binding free energy (ΔG_binding) calculations indicated that the Aa_P peptide derived from *A. albopictus* exhibited a more favorable binding energy than the hDSG3 EC1-derived peptide (**Table 4**), suggesting a high affinity for the risk-associated HLA-DRB1*04:02 molecule. Structural similarity and comparable binding modes support the potential for molecular mimicry (**Figures 2**, **3**). These computational results provide preliminary support for the VCM hypothesis and guided the development of a mechanistic model (**Figure 5**).

## 4 Discussion

Pemphigus vulgaris (PV) is a potentially life-threatening autoimmune blistering disease, and the mechanisms underlying the development of autoantibody responses against desmogleins remain incompletely understood. The disease is widely considered to arise from the interplay between genetic susceptibility and environmental factors, with human leukocyte antigen (HLA) class II genes representing the strongest and most consistently replicated genetic contributors. Multiple independent cohort studies have implicated specific *HLA-DRB1* alleles, particularly HLA-DRB1*04:02 and HLA-DRB1*14:01, as being associated with increased susceptibility to PV (29, 30). The prevalence of HLA-DRB1*04:02 has been reported to range from approximately 33% to over 90% across different PV populations, while HLA-DRB1*14:01 has been observed in 28–44% of patients in several cohorts (30).

In the present study, HLA-DRB1*04:02 and HLA-DRB1*14:01 were detected in 40.7% and 18% of PV patients, respectively, findings that are broadly consistent with previously reported ranges. The observed frequency of HLA-DRB1*14:01 in our cohort was lower than that reported in several other populations (**Table 1**). In contrast, HLA-DRB1*16:01 and HLA-DRB1*11:01 were less frequent among PV patients than in controls, suggesting a possible inverse association with disease susceptibility in this cohort (**Table 2**). Previous studies investigating HLA associations in PV have predominantly relied on allele- or haplotype-based analyses (31–36). In the present cohort, genotype-level analysis revealed that the heterozygous HLA-DRB1*04:02/14:01 genotype was observed exclusively among PV patients and not detected in controls. This distribution resulted in a high odds ratio estimate (OR: 114.97; 95% CI: 6.86–1925.82); however, the wide confidence interval reflects the low frequency of this genotype and indicates substantial statistical uncertainty (**Table 3**). Unlike typical genetic association studies that focus on common bi-allelic single-nucleotide polymorphisms (SNPs) (37), *HLA-DRB1* is highly polymorphic, comprising hundreds of distinct alleles. This extensive allelic diversity gives rise to numerous rare genotype combinations, which substantially limits statistical power in small- to moderate-sized cohorts. For this reason, most previous studies have emphasized allele- or haplotype-level associations rather than genotype-level patterns. While our findings suggest a notable enrichment of a specific heterozygous genotype within this cohort, confirmation of genotype-level associations will require evaluation in larger and independent populations with sufficient genotype counts.

Collectively, these observations reinforce the central role of *HLA-DRB1* variation in PV susceptibility while underscoring that genetic predisposition alone is unlikely to be sufficient for disease development. A range of environmental and microbial factors—including diet, medications, and infections—has been proposed as potential contributors to disease initiation (5, 38–40), yet none has been conclusively shown to account for the initial breach of immune tolerance. In the context of endemic pemphigus foliaceus (Fogo selvagem), exposure to salivary antigens from hematophagous insects, such as sandflies and blackflies, has been proposed as a possible environmental contributor to the development of anti-DSG1 autoantibodies (13, 14, 39). Building on this conceptual framework, and to explore the plausibility of the proposed VCM hypothesis, we applied an integrative *in silico* approach combining HLA genotyping, peptide-binding prediction (**Figure 1**, **Supplementary File**), structural modeling (**Figures 2**, **3**), and molecular dynamics simulations (**Table 4**). These analyses were designed to assess whether cadherin-like proteins derived from hematophagous vectors could exhibit structural and immunological similarity to hDSG3 that may be relevant in individuals carrying PV-associated *HLA-DRB1* alleles. We propose that repeated exposure to hematophagous vectors may represent a plausible environmental context in which cadherin-like proteins originating from salivary gland epithelial cells become immunologically relevant to the host in genetically predisposed individuals (**Figure 5**). The salivary glands of *A. albopictus* consist of a single layer of secretory epithelial cells, typically cuboidal or columnar, organized into tubular acini and ducts (41). As in other epithelial tissues, these cells express cadherins at their apicolateral membranes to maintain epithelial integrity and polarity (42, 43). During blood feeding, mechanical stress, physiological cell turnover, or cellular damage associated with apoptosis, necrosis, or infection could allow membrane-associated cadherin-like fragments to become accessible within the salivary milieu. While the presence of cadherin transcripts (**Supplementary Table S3**) and epithelial integrity mechanisms in mosquito salivary glands is well supported, direct evidence for the abundance, stability, and immunogenicity of cadherin-derived proteins within mosquito saliva remains limited. Accordingly, this proposed pathway is presented as a biologically plausible, yet hypothesis-generating model rather than a demonstrated pathogenic mechanism.

### 4.1 Conceptual framework: from vector-associated immune priming to desmoglein-targeted autoimmunity

In genetically susceptible individuals (e.g., carriers of HLA-DRB1*04:02 or *14:01), repeated exposure to cadherin-like peptide candidates potentially present in the saliva of *Aedes albopictus* or other hematophagous insects (e.g., sandflies) may be associated with a subclinical or priming immune response against these foreign antigens. One plausible scenario involves immune priming during vector bites, in which tissue injury or microbial co-inoculation generates damage-associated molecular patterns (DAMPs) or pathogen-associated molecular patterns (PAMPs). These signals may promote dendritic cell activation and favor the differentiation of naïve T cells toward immunogenic rather than tolerogenic phenotypes. In the presence of sequence and structural similarity to hDSG3 and hDSG1, certain B- and T-cell clones may therefore exhibit features consistent with molecular mimicry and potential cross-reactivity. Over time, somatic hypermutation, affinity maturation, and class-switch recombination within germinal centers (44) may enhance the specificity and binding strength of these B-cell receptors, potentially increasing the likelihood of recognition of self-antigens (DSG3/DSG1) (45). Initial tissue damage may further expose cryptic desmoglein epitopes, which are processed and presented by antigen-presenting cells, thereby promoting epitope spreading. This process could drive clonal expansion of autoreactive B cells and facilitate a transition from vector-derived immune recognition to sustained autoimmunity. Ultimately, persistent autoantibody production against DSG3 and DSG1 could contribute to disruption of keratinocyte adhesion, consistent with mechanisms underlying blister formation in pemphigus vulgaris (**Figure 5**).

To test our VCM hypothesis, at least *in silico*, we employed a combination of generating HLA-DRA/DRB1*04:02 complexes, peptide-binding predictions, structural modeling, and molecular dynamics analyses (**Table 4**). Our analysis revealed that the Aa_P peptide exhibited lower estimated binding free energy than the hDSG3 EC1-derived peptide in our *in silico* analyses. These data provide preliminary support for the VCM hypothesis, although experimental validation is required. Additional contextual support for the plausibility of the hypothesis comes from transcriptomic data reported by Sim and colleagues, indicating regulation of cadherin-related transcripts in the salivary glands of *Aedes aegypti* (46) (**Supplementary Table S3**). For experimental validation, an appropriate animal model is essential. Humanized mice expressing the HLA-DRB1*04:02 allele can be utilized, as previously demonstrated by Eming et al. in a preclinical mouse model of PV (47). The VCM hypothesis could be conceptually evaluated in future studies using these mice with both recombinant hDSG3 ectodomains and *A. albopictus*–derived cadherin-like (CAD-like) peptides (**Figures 1A**, **2B**).

### 4.2 Vector-associated mimicry in pemphigus: lessons from endemic pemphigus foliaceus and implications for pemphigus vulgaris

In regions such as Brazil and Tunisia, endemic pemphigus foliaceus (EPF)—including its localized form Fogo Selvagem (FS)—has long been suspected to arise from environmental exposures, particularly to insect vectors. Multiple studies have identified associations between anti-DSG1 autoantibodies and exposure to vector-borne antigens. For example, in Brazil, *Lutzomyia longipalpis* (the sandfly vector of visceral leishmaniasis) was shown to elicit anti-Dsg1 IgG4 antibodies through salivary proteins such as LJM11 and LJM17, which cross-react with epitopes on human Dsg1. In murine models, immunization with LJM11 induced anti-Dsg1 antibodies, lending support to the hypothesis of vector-induced molecular mimicry in genetically susceptible individuals (e.g., HLA-DRB1*04:04 carriers) (12–15). Similarly, in Tunisia, anti-Dsg1 antibodies have been detected in sera of patients with leishmaniasis and hydatidosis (48). Salivary proteins such as PpSP32 from *Phlebotomus papatasi* were also shown to bind both Dsg1 and Dsg3 *in vitro*. Interestingly, although vector antigens triggered desmoglein-recognizing antibodies in mice, definitive cross-reactivity and pathogenic potential remain unconfirmed. These observations suggest a possible mimicry mechanism but fall short of providing direct evidence for pathogenic antibody induction and acantholysis (13, 14, 48, 49). While these studies contribute valuable epidemiological and immunological insights, they do not fully explain the molecular mechanisms of epitope recognition or autoimmune activation. Notably, sandfly-derived proteins studied thus far have not been conclusively shown to generate immunodominant or acantholytic responses, and structural mimicry has not been conclusively demonstrated through high-resolution computational or crystallographic methods (48, 50). Against this backdrop, our current study advances the field by focusing not on salivary proteins per se, but rather on cadherin-like proteins encoded by the vectors themselves, particularly those with structural homology to the EC1 domain of human Dsg3—the primary autoantigen in PV. This novel approach addresses a major gap in the literature by proposing that vector-derived cadherin homologs may act as molecular mimics of hDsg3. Unlike earlier research, our study integrates peptide–HLA binding predictions, homology modeling, and molecular dynamics simulations to explore the plausibility of cross-reactive interactions. By centering on HLA-DRB1*04:02, a well-established susceptibility allele for PV, we propose a mechanistically and structurally coherent framework for how vector-derived proteins may break immune tolerance and initiate autoimmunity in genetically predisposed individuals.

### 4.3 Limitations

Several limitations of the present study should be acknowledged. First, although statistically significant associations were observed for HLA-DRB1*04:02 and HLA-DRB1*14:01 alleles, genotype-level analyses—particularly for the heterozygous HLA-DRB1*04:02/14:01 genotype—were based on low-frequency observations. As a consequence, estimates of effect size for rare genotypes are subject to statistical uncertainty and imprecision, and confidence intervals should be interpreted with caution. Under conditions of sparse data, odds ratio estimates may be inflated and do not necessarily reflect stable population-level effects.

Second, the study cohort size, while sufficient to detect allele-level associations, limits the robustness of genotype-level inference. Confirmation of the observed genotype-level enrichment will therefore require replication in larger, independent cohorts and in populations with different genetic backgrounds.

Third, the molecular dynamics simulations and binding free energy analyses were conducted over a finite simulation timescale and were designed for comparative, hypothesis-generating purposes. These *in silico* analyses do not provide direct evidence of pathogenicity, immunogenicity, or causal involvement in disease initiation, and should be interpreted as supportive structural and energetic observations rather than definitive mechanistic proof.

Finally, the proposed Vector-derived Cadherin Mimicry (VCM) framework is conceptual in nature. While it integrates immunogenetic associations with structural modeling, experimental validation—using appropriate cellular or animal models—will be necessary to assess the biological relevance of this hypothesis and its potential contribution to pemphigus vulgaris pathogenesis.

In conclusion, while vector-associated molecular mimicry has been most extensively explored in the context of endemic pemphigus foliaceus, the present study proposes a computationally informed and structurally plausible extension of this conceptual framework to PV, with a shift in focus from DSG1 to DSG3. Rather than establishing a definitive mechanistic link, this hypothesis expands existing models of vector-associated autoimmunity by integrating immunogenetic context with structural bioinformatics. To the best of our knowledge, this study also reports a significant enrichment of the heterozygous HLA-DRB1*04:02/14:01 genotype in a PV cohort, although confirmation of genotype-level effects will require validation in larger, independent populations. In parallel, our analyses suggest a previously underexplored connection between genetic susceptibility and repeated environmental exposure to vector-derived cadherin-like proteins. Through the integration of immunogenetic data, structural modeling, and comparative energetic analyses, we provide preliminary *in silico* observations consistent with the proposed VCM framework. If supported by future experimental studies, this model could contribute to a broader understanding of autoimmune pathogenesis, in which hematophagous insect vectors may represent context-dependent environmental factors rather than direct causal agents. Such insights may ultimately inform HLA-based risk stratification approaches and guide the development of targeted preventive strategies in genetically susceptible populations.

## Data Availability

The datasets presented in this study can be found in online repositories. The names of the repository/repositories and accession number(s) can be found below: https://doi.org/10.5281/zenodo.17593045, https://doi.org/10.5281/zenodo.17593045.

## References

[1] Amagai M Stanley JR . Desmoglein as a Target in Skin Disease and Beyond. J Invest Dermatol. (2012) 132:776–84. doi: 10.1038/jid.2011.390, PMID: 22189787 PMC3279627

[2] Stanley JR Amagai M . Pemphigus, Bullous Impetigo, and the Staphylococcal Scalded-Skin Syndrome. N Engl J Med. (2006) 355:1800–10. doi: 10.1056/NEJMra061111, PMID: 17065642

[3] Amagai M . The molecular logic of pemphigus and impetigo: the desmoglein story. Vet Dermatol. (2009) 20:308–12. doi: 10.1111/j.1365-3164.2009.00831.x, PMID: 20178466

[4] Payne AS Hanakawa Y Amagai M Stanley JR . Desmosomes and disease: pemphigus and bullous impetigo. Curr Opin Cell Biol. (2004) 16:536–43. doi: 10.1016/j.ceb.2004.07.006, PMID: 15363804

[5] Moro F Sinagra JLM Salemme A Fania L Mariotti F Pira A . Pemphigus: trigger and predisposing factors. Front Med. (2023) 10:1326359. doi: 10.3389/fmed.2023.1326359, PMID: 38213911 PMC10783816

[6] Didona D Di Zenzo G . Humoral Epitope Spreading in Autoimmune Bullous Diseases. Front Immunol. (2018) 9:779. doi: 10.3389/fimmu.2018.00779, PMID: 29719538 PMC5913575

[7] Tangye SG Ma CS Brink R Deenick EK . The good, the bad and the ugly — TFH cells in human health and disease. Nat Rev Immunol. (2013) 13:412–26. doi: 10.1038/nri3447, PMID: 23681096

[8] Vanderlugt CL Miller SD . Epitope spreading in immune-mediated diseases: implications for immunotherapy. Nat Rev Immunol. (2002) 2:85–95. doi: 10.1038/nri724, PMID: 11910899

[9] Feng X Zheng H Wang M Wang Y Zhou X Zhang X . Autoimmune bullous diseases: pathogenesis and clinical management. Mol BioMed. (2025) 6:30. doi: 10.1186/s43556-025-00272-9, PMID: 40372624 PMC12081819

[10] Tron F Gilbert D Mouquet H Joly P Drouot L Makni S . Genetic factors in pemphigus. J Autoimmun. (2005) 24:319–28. doi: 10.1016/j.jaut.2005.03.006, PMID: 15869862

[11] Baker J Seiffert-Sinha K Sinha AA . Patient genetics shape the autoimmune response in the blistering skin disease pemphigus vulgaris. Front Immunol. (2023) 13:1064073. doi: 10.3389/fimmu.2022.1064073, PMID: 36703961 PMC9871500

[12] Vodo D Sprecher E . The genetic basis of pemphigus vulgaris. JEADV Clin Pract. (2023) 2:203–12. doi: 10.1002/jvc2.129

[13] Aoki V Huang MHT Périgo AM Fukumori LMI Maruta CW Santi CG . Endemic pemphigus foliaceus (fogo selvagem) and pemphigus vulgaris: immunoglobulin G heterogeneity detected by indirect immunofluorescence. Rev Hosp Clínicas. (2004) 59:251–6. doi: 10.1590/S0041-87812004000500005, PMID: 15543395

[14] Hans-Filho G Aoki V Bittner NRH Bittner GC . Fogo selvagem: endemic pemphigus foliaceus. Bras Dermatol. (2018) 93:638–50. doi: 10.1590/abd1806-4841.20188235, PMID: 30156612 PMC6106655

[15] Eaton DP Diaz LA Hans-Filho G Santos VD Aoki V Friedman H . Comparison of Black Fly Species (Diptera: Simuliidae) on an Amerindian Reservation with a High Prevalence of Fogo Selvagem to Neighboring Disease-Free Sites in the State of Mato Grosso do Sul, Brazil. J Med Entomol. (1998) 35:120–31. doi: 10.1093/jmedent/35.2.120, PMID: 9538571

[16] Itoh Y Mizuki N Shimada T Azuma F Itakura M Kashiwase K . High-throughput DNA typing of HLA-A, -B, -C, and -DRB1 loci by a PCR–SSOP–Luminex method in the Japanese population. Immunogenetics. (2005) 57:717–29. doi: 10.1007/s00251-005-0048-3, PMID: 16215732

[17] Testi M Iannelli S Testa G Troiano M Capelli S Fruet F . Evaluation of DRB1 high resolution typing by a new SSO-based Luminex method. Mol Biol Rep. (2012) 39:13–6. doi: 10.1007/s11033-011-0704-7, PMID: 21424786

[18] Menashe I Rosenberg PS Chen BE . PGA: power calculator for case-control genetic association analyses. BMC Genet. (2008) 9:36. doi: 10.1186/1471-2156-9-36, PMID: 18477402 PMC2387159

[19] Rousset F . genepop’007: a complete re-implementation of the genepop software for Windows and Linux. Mol Ecol Resour. (2008) 8:103–6. doi: 10.1111/j.1471-8286.2007.01931.x, PMID: 21585727

[20] Altschul S. Gapped BLAST . and PSI-BLAST: a new generation of protein database search programs. Nucleic Acids Res. (1997) 25:3389–402. doi: 10.1093/nar/25.17.3389, PMID: 9254694 PMC146917

[21] Schaffer AA . Improving the accuracy of PSI-BLAST protein database searches with composition-based statistics and other refinements. Nucleic Acids Res. (2001) 29:2994–3005. doi: 10.1093/nar/29.14.2994, PMID: 11452024 PMC55814

[22] Abramson J Adler J Dunger J Evans R Green T Pritzel A . Accurate structure prediction of biomolecular interactions with AlphaFold 3. Nature. (2024) 630:493–500. doi: 10.1038/s41586-024-07487-w, PMID: 38718835 PMC11168924

[23] Omidi A Møller MH Malhis N Bui JM Gsponer J . AlphaFold-Multimer accurately captures interactions and dynamics of intrinsically disordered protein regions. Proc Natl Acad Sci. (2024) 121:e2406407121. doi: 10.1073/pnas.2406407121, PMID: 39446390 PMC11536093

[24] Mirdita M Schütze K Moriwaki Y Heo L Ovchinnikov S Steinegger M . ColabFold: making protein folding accessible to all. Nat Methods. (2022) 19:679–82. doi: 10.1038/s41592-022-01488-1, PMID: 35637307 PMC9184281

[25] Meng EC Goddard TD Pettersen EF Couch GS Pearson ZJ Morris JH . UCSF ChimeraX: Tools for structure building and analysis. Protein Sci. (2023) 32:e4792. doi: 10.1002/pro.4792, PMID: 37774136 PMC10588335

[26] Abraham MJ Murtola T Schulz R Páll S Smith JC Hess B . GROMACS: High performance molecular simulations through multi-level parallelism from laptops to supercomputers. SoftwareX. (2015) 1–2:19–25. doi: 10.1016/j.softx.2015.06.001

[27] Valdés-Tresanco MS Valdés-Tresanco ME Valiente PA Moreno E . gmx_MMPBSA: A New Tool to Perform End-State Free Energy Calculations with GROMACS. J Chem Theory Comput. (2021) 17:6281–91. doi: 10.1021/acs.jctc.1c00645, PMID: 34586825

[28] Miller BR McGee TD Swails JM Homeyer N Gohlke H Roitberg AE . MMPBSA.py : An Efficient Program for End-State Free Energy Calculations. J Chem Theory Comput. (2012) 8:3314–21. doi: 10.1021/ct300418h, PMID: 26605738

[29] Ahmed AR Yunis EJ Khatri K Wagner R Notani G Awdeh Z . Major histocompatibility complex haplotype studies in Ashkenazi Jewish patients with pemphigus vulgaris. Proc Natl Acad Sci. (1990) 87:7658–62. doi: 10.1073/pnas.87.19.7658, PMID: 2217197 PMC54807

[30] Panhuber A Lamorte G Bruno V Cetin H Bauer W Höftberger R . A systematic review and meta-analysis of HLA class II associations in patients with IgG4 autoimmunity. Sci Rep. (2022) 12:9229. doi: 10.1038/s41598-022-13042-2, PMID: 35654912 PMC9163138

[31] Lombardi ML Mercuro O Pirozzi G Manzo C Lombari V Ruocco V . Common Human Leukocyte Antigen Alleles in Pemphigus Vulgaris and Pemphigus Foliaceus Italian Patients. J Invest Dermatol. (1999) 113:107–10. doi: 10.1046/j.1523-1747.1999.00626.x, PMID: 10417627

[32] Veldman CM Gebhard KL Uter W Wassmuth R Grötzinger J Schultz E . T Cell Recognition of Desmoglein 3 Peptides in Patients with Pemphigus Vulgaris and Healthy Individuals. J Immunol. (2004) 172:3883–92. doi: 10.4049/jimmunol.172.6.3883, PMID: 15004196

[33] Yan L Wang J Zeng K . Association between HLA-DRB1 polymorphisms and pemphigus vulgaris: a meta-analysis. Br J Dermatol. (2012) 167:768–77. doi: 10.1111/j.1365-2133.2012.11040.x, PMID: 22564118 PMC3485671

[34] Shams S Amirzargar AA Yousefi M Rezaei N Solgi G Khosravi F . HLA Class II (DRB, DQA1 and DQB1) Allele and Haplotype Frequencies in the Patients with Pemphigus Vulgaris. J Clin Immunol. (2009) 29:175–9. doi: 10.1007/s10875-008-9244-x, PMID: 18780165

[35] Miyagawa S Higashimine I Iida T Yamashina Y Fukumoto T Shirai T . HLA-DRB1*04 and DRB1*14 Alleles Are Associated with Susceptibility to Pemphigus Among Japanese. J Invest Dermatol. (1997) 109:615–8. doi: 10.1111/1523-1747.ep12337585, PMID: 9347787

[36] Saha M Harman K Mortimer NJ Binda V Black MM Kondeatis E . Pemphigus Vulgaris in White Europeans Is Linked with HLA Class II Allele HLA DRB1*1454 but Not DRB1*1401. J Invest Dermatol. (2010) 130:311–4. doi: 10.1038/jid.2009.241, PMID: 19847191

[37] Clarke GM Anderson CA Pettersson FH Cardon LR Morris AP Zondervan KT . Basic statistical analysis in genetic case-control studies. Nat Protoc. (2011) 6:121–33. doi: 10.1038/nprot.2010.182, PMID: 21293453 PMC3154648

[38] Stone C Bak G Oh D Zhao C Venugopal S Kumar K . Environmental triggers of pemphigus vulgaris and bullous pemphigoid: a case control study. Front Med. (2024) 11:1441369. doi: 10.3389/fmed.2024.1441369, PMID: 39502648 PMC11537152

[39] Qian Y Culton DA Jeong JS Trupiano N Valenzuela JG Diaz LA . Non-infectious environmental antigens as a trigger for the initiation of an autoimmune skin disease. Autoimmun Rev. (2016) 15:923–30. doi: 10.1016/j.autrev.2016.07.005, PMID: 27396816 PMC4982806

[40] Ruocco V Ruocco E Lo Schiavo A Brunetti G Guerrera LP Wolf R . Pemphigus: Etiology, pathogenesis, and inducing or triggering factors: Facts and controversies. Clin Dermatol. (2013) 31:374–81. doi: 10.1016/j.clindermatol.2013.01.004, PMID: 23806154

[41] Barreau C Conrad J Fischer E Lujan HD Vernick KD . Identification of surface molecules on salivary glands of the mosquito, Aedes aegypti, by a panel of monoclonal antibodies. Insect Biochem Mol Biol. (1999) 29:515–26. doi: 10.1016/S0965-1748(99)00025-9, PMID: 10406090

[42] Alves e Silva TL Joseph RE Vega-Rodriguez J . Beyond the bite: how mosquito salivary proteins modulate midgut biology and malaria parasite transmission. Curr Opin Insect Sci. (2025) 69:101363. doi: 10.1016/j.cois.2025.101363, PMID: 40081801 PMC12066222

[43] Juhn J Naeem-Ullah U Maciel Guedes BA Majid A Coleman J Paolucci Pimenta PF . Spatial mapping of gene expression in the salivary glands of the dengue vector mosquito, Aedes aegypti. Parasit Vectors. (2011) 4:1. doi: 10.1186/1756-3305-4-1, PMID: 21205315 PMC3043528

[44] Liu J Zhang K Zhang X Guan F Zeng H Kubo M . Immunoglobulin class-switch recombination: Mechanism, regulation, and related diseases. MedComm. (2024) 5:e662. doi: 10.1002/mco2.662, PMID: 39144468 PMC11322596

[45] Inoue T Baba Y Kurosaki T . BCR signaling in germinal center B cell selection. Trends Immunol. (2024) 45:693–704. doi: 10.1016/j.it.2024.07.005, PMID: 39168721

[46] Sim S Ramirez JL Dimopoulos G . Dengue Virus Infection of the Aedes aegypti Salivary Gland and Chemosensory Apparatus Induces Genes that Modulate Infection and Blood-Feeding Behavior. PloS Pathog. (2012) 8:e1002631. doi: 10.1371/journal.ppat.1002631, PMID: 22479185 PMC3315490

[47] Eming R Hennerici T Bäcklund J Feliciani C Visconti KC Willenborg S . Pathogenic IgG Antibodies against Desmoglein 3 in Pemphigus Vulgaris Are Regulated by HLA-DRB1*04:02–Restricted T Cells. J Immunol. (2014) 193:4391–9. doi: 10.4049/jimmunol.1401081, PMID: 25252957

[48] Zaraa I Boussoffara T Ben Ahmed M Marzouki S Ben Hassouna N Kallel Sellami M . Exposure to *Phlebotomus papatasi* and/or *Leishmania major*: Possible etiologic link to Tunisian pemphigus. Clin Exp Dermatol. (2011) 36:845–50. doi: 10.1111/j.1365-2230.2011.04140.x, PMID: 22011908

[49] Aoki V Abdeladhim M Li N Cecilio P Prisayanh P Diaz LA . Some Good and Some Bad: Sand Fly Salivary Proteins in the Control of Leishmaniasis and in Autoimmunity. Front Cell Infect Microbiol. (2022) 12:839932. doi: 10.3389/fcimb.2022.839932, PMID: 35281450 PMC8913536

[50] Li N Aoki V Liu Z Prisayanh P Valenzuela JG Qian Y . From insect bites to a skin autoimmune disease: a conceivable pathway to endemic pemphigus foliaceus. Front Immunol. (2022) 13:907424. doi: 10.3389/fimmu.2022.907424, PMID: 35693761 PMC9186141

