# Supplementary material for: Could vector-derived cadherin mimicry contribute to pemphigus vulgaris? An immunogenetic and in silico study involving HLA-DRB104:02 and 14:01
Source: Front Immunol. 2026 Feb 18;17:1745207. doi: 10.3389/fimmu.2026.1745207 (PMC12956788; doi:10.3389/fimmu.2026.1745207)

**SUPPLEMENTARY MATERIAL**

**Supplementary Methods and Results**

**SUPPLEMENTARY METHODS (SM)**

**SM1. Sequence Retrieval and Similarity Analysis**

Protein sequences corresponding to cadherin-like proteins from *Aedes albopictus* were retrieved from VectorBase (https://vectorbase.org). Sequence similarity searches were performed using BLASTp (v2.10.1+) against curated arthropod protein databases to identify cadherin-like domains and to exclude closely related human homologs. Default BLASTp parameters were used unless otherwise specified. Only *A. albopictus* sequences were included in downstream analyses to maintain species specificity. Candidate regions showing cadherin-like features were further inspected for the presence of extracellular cadherin (EC1) domains based on domain annotations and sequence composition. Regions corresponding to EC1-like domains were extracted for subsequent peptide prediction and structural analyses.

**SM2. Peptide Selection and Similarity Assessment**

Cadherin-like EC1 domain fragments identified from *A. albopictus* proteins were subjected to MHC class II peptide-binding prediction using the IEDB analysis resource. Peptides were evaluated for predicted binding affinity to HLA-DRB1*04:02 and HLA-DRB1*14:01 alleles using the recommended consensus prediction approach. Peptides with higher predicted binding ranks were prioritized for further analysis. To assess similarity with human desmogleins, candidate vector-derived peptides were compared with peptides derived from the EC1 domain of human DSG3. Similarity assessment was based on a combination of sequence features, physicochemical properties, and structural context rather than strict sequence identity. Peptides showing partial motif overlap or compatible residue patterns with DSG3 EC1-derived peptides were selected for structural modeling and molecular dynamics analyses. This prioritization strategy was designed to identify peptides with plausible cross-reactive potential while avoiding assumptions regarding direct immunogenicity or pathogenicity.

**SM3. Molecular Dynamics Simulation Details**

Molecular dynamics (MD) simulations were performed using GROMACS (version 2022.5) with the AMBER99SB-ILDN force field to model peptide–HLA class II complexes. Initial peptide–HLA structures were embedded in a rectangular simulation box and solvated with TIP3P explicit water molecules, maintaining a minimum distance of 1.0 nm between any protein atom and the box boundary. System neutrality was achieved by adding appropriate counterions (Na⁺ or Cl⁻). Energy minimization was carried out using the steepest descent algorithm until convergence, defined as a maximum force below 1000 kJ·mol⁻¹·nm⁻¹. Following minimization, systems were equilibrated in two sequential phases:(i) NVT equilibration to stabilize temperature at 298 K using the V-rescale thermostat, and (ii) NPT equilibration to stabilize pressure at 1 atm using the Parrinello–Rahman barostat, allowing isotropic box scaling. Production MD simulations were subsequently performed for 100 ns under NPT conditions with a time step of 2 fs. Long-range electrostatic interactions were treated using the particle mesh Ewald (PME) method with a real-space cutoff of 1.0 nm. Van der Waals interactions were truncated at the same cutoff distance. All covalent bonds involving hydrogen atoms were constrained using the LINCS algorithm, enabling stable integration at the chosen time step. Periodic boundary conditions were applied in all spatial dimensions. Trajectory frames were saved at regular intervals for subsequent structural, dynamic, and energetic analyses. These simulation settings were selected to ensure stable sampling of peptide–HLA interactions while maintaining consistency across all analyzed systems.

**SM4. RMSD and RMSF Analyses**

Trajectory analyses were performed to assess peptide stability and residue-level flexibility within the HLA binding groove using a combination of GROMACS-based tools and Python-based trajectory analysis workflows implemented in a Google Colab environment. All trajectories were first processed to remove periodic boundary condition artifacts and to ensure consistent atom indexing. For RMSD calculations, peptide backbone atoms were analyzed after least-squares alignment of each trajectory frame to the backbone atoms of the HLA molecule. This alignment strategy was used to minimize contributions from global translational and rotational motion of the complex and to isolate peptide-specific conformational dynamics within the HLA binding groove. Peptide backbone root mean square deviation (RMSD) computed using both GROMACS utilities (gmx rms) and Python-based analysis implemented with the MDAnalysis library. RMSD time series were evaluated to confirm convergence and stability of peptide conformations during the production phase of the simulations. Residue-level flexibility was assessed by calculating root mean square fluctuation (RMSF) values for individual peptide residues. RMSF calculations were performed using MDAnalysis by evaluating positional fluctuations of peptide backbone atoms around their time-averaged coordinates following HLA-based alignment. This approach is conceptually equivalent to GROMACS-based RMSF calculations (gmx rmsf) while allowing seamless integration with downstream Python-based visualization and data processing. To ensure consistency across all simulated systems and to exclude early equilibration effects, only the final 47.5 ns of each 100 ns production trajectory were included in RMSD and RMSF analyses. This equilibrated window was selected based on inspection of RMSD convergence behavior across trajectories. RMSD and RMSF results were used to provide comparative and descriptive measures of peptide dynamics within the HLA binding groove. These analyses were not intended to infer functional immunogenicity, pathogenicity, or binding affinity, but rather to characterize relative stability and flexibility patterns across peptide–HLA complexes under consistent simulation conditions. All RMSD and RMSF plots were generated within a Google Colab environment using Python-based visualization tools. Time-series RMSD plots and residue-wise RMSF profiles were produced using Matplotlib for graphical rendering, with numerical data handling performed using NumPy and Pandas where appropriate. Plots were generated directly from MDAnalysis-derived arrays to ensure consistency between numerical analysis and visualization. Axis scaling, labeling, and color schemes were kept uniform across peptide systems to facilitate direct comparison. No smoothing or post hoc data manipulation was applied beyond standard averaging procedures inherent to RMSF calculations. All figures presented in the Supplementary Results were intended for descriptive and comparative visualization of peptide dynamics and were not used to derive statistical inference or functional conclusions.

**SM5. Binding Free Energy Calculations**

Binding free energies (ΔGbinding) for peptide–HLA complexes were estimated using the gmx_MM/PBSA package (version 1.5.7) employing the MM/GBSA approach with the GB-OBC II implicit solvent model (igb = 5). A single-trajectory protocol was used, in which complex, receptor, and ligand energies were derived from the same molecular dynamics trajectory. Energy calculations were performed on the final frames of the equilibrated trajectories. Per-residue energy decomposition was conducted to identify residues contributing to peptide–HLA interactions. These energetic estimates were used for relative comparison between peptide systems and were not interpreted as absolute binding free energies.

**SUPPLEMENTARY RESULTS (SR)**

**SR1. Sequence Retrieval and Similarity Analysis**

BLASTP 2.10.1+

Reference: Stephen F. Altschul, Thomas L. Madden, Alejandro A.

Schaffer, Jinghui Zhang, Zheng Zhang, Webb Miller, and David J.

Lipman (1997), "Gapped BLAST and PSI-BLAST: a new generation of

protein database search programs", Nucleic Acids Res. 25:3389-3402.

Reference for composition-based statistics: Alejandro A. Schaffer,

L. Aravind, Thomas L. Madden, Sergei Shavirin, John L. Spouge, Yuri

I. Wolf, Eugene V. Koonin, and Stephen F. Altschul (2001),

"Improving the accuracy of PSI-BLAST protein database searches with

composition-based statistics and other refinements", Nucleic Acids

Res. 29:2994-3005.

Database: /eupath/data/apiSiteFilesStaging/VectorBase/49/real/webServices/Vect

orBase/release-

CURRENT/AaegyptiLVP_AGWG/blast/AaegyptiLVP_AGWGAnnotatedProteins;

/eupath/data/apiSiteFilesStaging/VectorBase/49/real/webServices/Vect

orBase/release-

CURRENT/AalbimanusSTECLA2020/blast/AalbimanusSTECLA2020AnnotatedProt

eins;

/eupath/data/apiSiteFilesStaging/VectorBase/49/real/webServices/Vect

orBase/release-

CURRENT/AalbimanusSTECLA/blast/AalbimanusSTECLAAnnotatedProteins;

/eupath/data/apiSiteFilesStaging/VectorBase/49/real/webServices/Vect

orBase/release-CURRENT/AalbopictusC6-36/blast/AalbopictusC6-

36AnnotatedProteins;

/eupath/data/apiSiteFilesStaging/VectorBase/49/real/webServices/Vect

orBase/release-

CURRENT/AalbopictusFoshan/blast/AalbopictusFoshanAnnotatedProteins;

/eupath/data/apiSiteFilesStaging/VectorBase/49/real/webServices/Vect

orBase/release-

CURRENT/AalbopictusFoshanFPA/blast/AalbopictusFoshanFPAAnnotatedProt

eins;

/eupath/data/apiSiteFilesStaging/VectorBase/49/real/webServices/Vect

orBase/release-

CURRENT/AaquasalisAaquGF1/blast/AaquasalisAaquGF1AnnotatedProteins;

/eupath/data/apiSiteFilesStaging/VectorBase/49/real/webServices/Vect

orBase/release-

CURRENT/AarabiensisDONGOLA2021/blast/AarabiensisDONGOLA2021Annotated

Proteins;

/eupath/data/apiSiteFilesStaging/VectorBase/49/real/webServices/Vect

orBase/release-

CURRENT/AarabiensisDongola/blast/AarabiensisDongolaAnnotatedProteins

;

/eupath/data/apiSiteFilesStaging/VectorBase/49/real/webServices/Vect

orBase/release-

CURRENT/AatroparvusEBRO/blast/AatroparvusEBROAnnotatedProteins;

/eupath/data/apiSiteFilesStaging/VectorBase/49/real/webServices/Vect

orBase/release-

CURRENT/AbellatorAbelBR1/blast/AbellatorAbelBR1AnnotatedProteins;

/eupath/data/apiSiteFilesStaging/VectorBase/49/real/webServices/Vect

orBase/release-

CURRENT/AchristyiACHKN1017/blast/AchristyiACHKN1017AnnotatedProteins

;

/eupath/data/apiSiteFilesStaging/VectorBase/49/real/webServices/Vect

orBase/release-

CURRENT/AcoluzziiAcolN3/blast/AcoluzziiAcolN3AnnotatedProteins;

/eupath/data/apiSiteFilesStaging/VectorBase/49/real/webServices/Vect

orBase/release-

CURRENT/AcoluzziiMOPTI/blast/AcoluzziiMOPTIAnnotatedProteins;

/eupath/data/apiSiteFilesStaging/VectorBase/49/real/webServices/Vect

orBase/release-CURRENT/AcoluzziiMali-NIH/blast/AcoluzziiMali-

NIHAnnotatedProteins;

/eupath/data/apiSiteFilesStaging/VectorBase/49/real/webServices/Vect

orBase/release-

CURRENT/AcoluzziiNgousso/blast/AcoluzziiNgoussoAnnotatedProteins;

/eupath/data/apiSiteFilesStaging/VectorBase/49/real/webServices/Vect

orBase/release-

CURRENT/AcoustaniAcouGA1/blast/AcoustaniAcouGA1AnnotatedProteins;

/eupath/data/apiSiteFilesStaging/VectorBase/49/real/webServices/Vect

orBase/release-

CURRENT/AcruziiAcruBR1/blast/AcruziiAcruBR1AnnotatedProteins;

/eupath/data/apiSiteFilesStaging/VectorBase/49/real/webServices/Vect

orBase/release-CURRENT/AculicifaciesA-37/blast/AculicifaciesA-

37AnnotatedProteins;

/eupath/data/apiSiteFilesStaging/VectorBase/49/real/webServices/Vect

orBase/release-

CURRENT/AdarlingiAdarGF1/blast/AdarlingiAdarGF1AnnotatedProteins;

/eupath/data/apiSiteFilesStaging/VectorBase/49/real/webServices/Vect

orBase/release-

CURRENT/AdarlingiCoari/blast/AdarlingiCoariAnnotatedProteins;

/eupath/data/apiSiteFilesStaging/VectorBase/49/real/webServices/Vect

orBase/release-

CURRENT/AdirusWRAIR2/blast/AdirusWRAIR2AnnotatedProteins;

/eupath/data/apiSiteFilesStaging/VectorBase/49/real/webServices/Vect

orBase/release-

CURRENT/AepiroticusEpiroticus2/blast/AepiroticusEpiroticus2Annotated

Proteins;

/eupath/data/apiSiteFilesStaging/VectorBase/49/real/webServices/Vect

orBase/release-

CURRENT/AfarautiFAR1/blast/AfarautiFAR1AnnotatedProteins;

/eupath/data/apiSiteFilesStaging/VectorBase/49/real/webServices/Vect

orBase/release-

CURRENT/AfunestusAfunGA1/blast/AfunestusAfunGA1AnnotatedProteins;

/eupath/data/apiSiteFilesStaging/VectorBase/49/real/webServices/Vect

orBase/release-

CURRENT/AfunestusFUMOZ/blast/AfunestusFUMOZAnnotatedProteins;

/eupath/data/apiSiteFilesStaging/VectorBase/49/real/webServices/Vect

orBase/release-

CURRENT/AgambiaeIfakara/blast/AgambiaeIfakaraAnnotatedProteins;

/eupath/data/apiSiteFilesStaging/VectorBase/49/real/webServices/Vect

orBase/release-

CURRENT/AgambiaePEST/blast/AgambiaePESTAnnotatedProteins;

/eupath/data/apiSiteFilesStaging/VectorBase/49/real/webServices/Vect

orBase/release-

CURRENT/Amaculatusmaculatus3/blast/Amaculatusmaculatus3AnnotatedProt

eins;

/eupath/data/apiSiteFilesStaging/VectorBase/49/real/webServices/Vect

orBase/release-

CURRENT/AmaculipalpisAmacGA1/blast/AmaculipalpisAmacGA1AnnotatedProt

eins;

/eupath/data/apiSiteFilesStaging/VectorBase/49/real/webServices/Vect

orBase/release-

CURRENT/AmarshalliiAmarGA1/blast/AmarshalliiAmarGA1AnnotatedProteins

;

/eupath/data/apiSiteFilesStaging/VectorBase/49/real/webServices/Vect

orBase/release-

CURRENT/AmelasCM1001059_A/blast/AmelasCM1001059_AAnnotatedProteins;

/eupath/data/apiSiteFilesStaging/VectorBase/49/real/webServices/Vect

orBase/release-

CURRENT/AmerusMAF2021/blast/AmerusMAF2021AnnotatedProteins;

/eupath/data/apiSiteFilesStaging/VectorBase/49/real/webServices/Vect

orBase/release-CURRENT/AmerusMAF/blast/AmerusMAFAnnotatedProteins;

/eupath/data/apiSiteFilesStaging/VectorBase/49/real/webServices/Vect

orBase/release-

CURRENT/AminimusMINIMUS1/blast/AminimusMINIMUS1AnnotatedProteins;

/eupath/data/apiSiteFilesStaging/VectorBase/49/real/webServices/Vect

orBase/release-

CURRENT/AmouchetiAmouCM1/blast/AmouchetiAmouCM1AnnotatedProteins;

/eupath/data/apiSiteFilesStaging/VectorBase/49/real/webServices/Vect

orBase/release-

CURRENT/AniliAnilCM1/blast/AniliAnilCM1AnnotatedProteins;

/eupath/data/apiSiteFilesStaging/VectorBase/49/real/webServices/Vect

orBase/release-

CURRENT/AquadriannulatusSANGWE/blast/AquadriannulatusSANGWEAnnotated

Proteins;

/eupath/data/apiSiteFilesStaging/VectorBase/49/real/webServices/Vect

orBase/release-

CURRENT/AsinensisChina/blast/AsinensisChinaAnnotatedProteins;

/eupath/data/apiSiteFilesStaging/VectorBase/49/real/webServices/Vect

orBase/release-

CURRENT/AsinensisSINENSIS/blast/AsinensisSINENSISAnnotatedProteins;

/eupath/data/apiSiteFilesStaging/VectorBase/49/real/webServices/Vect

orBase/release-

CURRENT/AstephensiIndian/blast/AstephensiIndianAnnotatedProteins;

/eupath/data/apiSiteFilesStaging/VectorBase/49/real/webServices/Vect

orBase/release-CURRENT/AstephensiSDA-500/blast/AstephensiSDA-

500AnnotatedProteins;

/eupath/data/apiSiteFilesStaging/VectorBase/49/real/webServices/Vect

orBase/release-

CURRENT/AstephensiUCISS2018/blast/AstephensiUCISS2018AnnotatedProtei

ns;

/eupath/data/apiSiteFilesStaging/VectorBase/49/real/webServices/Vect

orBase/release-

CURRENT/AziemanniAzieGA1/blast/AziemanniAzieGA1AnnotatedProteins;

/eupath/data/apiSiteFilesStaging/VectorBase/49/real/webServices/Vect

orBase/release-

CURRENT/CquinquefasciatusJHB2020/blast/CquinquefasciatusJHB2020Annot

atedProteins;

/eupath/data/apiSiteFilesStaging/VectorBase/49/real/webServices/Vect

orBase/release-

CURRENT/CquinquefasciatusJohannesburg/blast/CquinquefasciatusJohanne

sburgAnnotatedProteins;

/eupath/data/apiSiteFilesStaging/VectorBase/49/real/webServices/Vect

orBase/release-

CURRENT/ScyaneusScyaPA1/blast/ScyaneusScyaPA1AnnotatedProteins

854,451 sequences; 547,869,893 total letters

Query=

Length=566

Score E

Sequences producing significant alignments: (Bits) Value

AALF007356-PA | transcript=AALF007356-RA | gene=AALF007356 | orga... 149 6e-36

ASIC022140-PA | transcript=ASIC022140-RA | gene=ASIC022140 | orga... 147 2e-35

AALB20_033171.P66527 | transcript=AALB20_033171.R66527 | gene=AAL... 147 2e-35

AATE014116-PA | transcript=AATE014116-RA | gene=AATE014116 | orga... 147 2e-35

AFUN2_006054.P9868 | transcript=AFUN2_006054.R9868 | gene=AFUN2_0... 147 2e-35

AALB20_033171.P66512 | transcript=AALB20_033171.R66512 | gene=AAL... 147 2e-35

AALB20_033171.P66523 | transcript=AALB20_033171.R66523 | gene=AAL... 147 2e-35

ACMO_007424.P13504 | transcript=ACMO_007424.R13504 | gene=ACMO_00... 147 2e-35

ADAR2_001930.P21642 | transcript=ADAR2_001930.R21642 | gene=ADAR2... 147 2e-35

AMAC_003346.P3008 | transcript=AMAC_003346.R3008 | gene=AMAC_0033... 147 2e-35

AALB20_033171.P66516 | transcript=AALB20_033171.R66516 | gene=AAL... 147 2e-35

AMEM21_003341.P6430 | transcript=AMEM21_003341.R6430 | gene=AMEM2... 147 2e-35

ASTEI20_040830.P55056 | transcript=ASTEI20_040830.R55056 | gene=A... 147 2e-35

AGAMI1_007196.P14118 | transcript=AGAMI1_007196.R14118 | gene=AGA... 147 2e-35

ACON2_034794.P62462 | transcript=ACON2_034794.R62462 | gene=ACON2... 147 2e-35

AAQUA_001764.P1522 | transcript=AAQUA_001764.R1522 | gene=AAQUA_0... 147 2e-35

AARA21_009090.P17043 | transcript=AARA21_009090.R17043 | gene=AAR... 147 2e-35

ADAR2_001930.P21645 | transcript=ADAR2_001930.R21645 | gene=ADAR2... 147 2e-35

AMEM21_003341.P6434 | transcript=AMEM21_003341.R6434 | gene=AMEM2... 147 2e-35

AARA21_009090.P17046 | transcript=AARA21_009090.R17046 | gene=AAR... 147 2e-35

AARA21_009090.P17034 | transcript=AARA21_009090.R17034 | gene=AAR... 147 2e-35

AMEM21_003341.P6428 | transcript=AMEM21_003341.R6428 | gene=AMEM2... 147 3e-35

AFUN2_006054.P9870 | transcript=AFUN2_006054.R9870 | gene=AFUN2_0... 147 3e-35

ASTEI20_040830.P55060 | transcript=ASTEI20_040830.R55060 | gene=A... 147 3e-35

AGAMI1_007196.P14115 | transcript=AGAMI1_007196.R14115 | gene=AGA... 147 3e-35

ACON2_034794.P62473 | transcript=ACON2_034794.R62473 | gene=ACON2... 147 3e-35

ACMO_007424.P13500 | transcript=ACMO_007424.R13500 | gene=ACMO_00... 147 3e-35

AGAMI1_007196.P14116 | transcript=AGAMI1_007196.R14116 | gene=AGA... 147 3e-35

ACON2_034794.P62470 | transcript=ACON2_034794.R62470 | gene=ACON2... 147 3e-35

ACON029589-PB | transcript=ACON029589-RB | gene=ACON029589 | orga... 147 3e-35

AALB20_033171.P66493 | transcript=AALB20_033171.R66493 | gene=AAL... 147 3e-35

AMEM21_003341.P6419 | transcript=AMEM21_003341.R6419 | gene=AMEM2... 147 3e-35

ACMO_007424.P13494 | transcript=ACMO_007424.R13494 | gene=ACMO_00... 147 3e-35

ASTEI20_040830.P55059 | transcript=ASTEI20_040830.R55059 | gene=A... 147 3e-35

AMEM21_003341.P6424 | transcript=AMEM21_003341.R6424 | gene=AMEM2... 147 3e-35

ACMO_007424.P13508 | transcript=ACMO_007424.R13508 | gene=ACMO_00... 147 3e-35

AARA21_009090.P17035 | transcript=AARA21_009090.R17035 | gene=AAR... 147 3e-35

AGAP029589-PB | transcript=AGAP029589-RB | gene=AGAP029589 | orga... 147 3e-35

AMAC_003346.P2999 | transcript=AMAC_003346.R2999 | gene=AMAC_0033... 147 3e-35

AMAC_003346.P3007 | transcript=AMAC_003346.R3007 | gene=AMAC_0033... 147 3e-35

ASTEI20_040830.P55062 | transcript=ASTEI20_040830.R55062 | gene=A... 147 3e-35

ASTEI20_040830.P55052 | transcript=ASTEI20_040830.R55052 | gene=A... 147 3e-35

ACMO_007424.P13496 | transcript=ACMO_007424.R13496 | gene=ACMO_00... 147 3e-35

AALB20_033171.P66511 | transcript=AALB20_033171.R66511 | gene=AAL... 147 3e-35

AARA21_009090.P17045 | transcript=AARA21_009090.R17045 | gene=AAR... 147 3e-35

AARA21_009090.P17036 | transcript=AARA21_009090.R17036 | gene=AAR... 147 3e-35

ADAR2_001930.P21644 | transcript=ADAR2_001930.R21644 | gene=ADAR2... 147 3e-35

ADAR2_001930.P21647 | transcript=ADAR2_001930.R21647 | gene=ADAR2... 147 3e-35

AALB20_033171.P66510 | transcript=AALB20_033171.R66510 | gene=AAL... 147 3e-35

AMAC_003346.P3003 | transcript=AMAC_003346.R3003 | gene=AMAC_0033... 147 3e-35

ASTEI20_040830.P55068 | transcript=ASTEI20_040830.R55068 | gene=A... 147 3e-35

AFUN2_006054.P9879 | transcript=AFUN2_006054.R9879 | gene=AFUN2_0... 147 3e-35

AFUN2_006054.P9862 | transcript=AFUN2_006054.R9862 | gene=AFUN2_0... 147 3e-35

AMEM21_003341.P6433 | transcript=AMEM21_003341.R6433 | gene=AMEM2... 146 4e-35

AGAMI1_007196.P14110 | transcript=AGAMI1_007196.R14110 | gene=AGA... 146 4e-35

ACMO_007424.P13509 | transcript=ACMO_007424.R13509 | gene=ACMO_00... 146 4e-35

ACON2_034794.P62457 | transcript=ACON2_034794.R62457 | gene=ACON2... 146 4e-35

AAEL000597-PB | transcript=AAEL000597-RB | gene=AAEL000597 | orga... 146 4e-35

AALFPA_070705.P41263 | transcript=AALFPA_070705.R41263 | gene=AAL... 146 5e-35

AALC636_024560.P34471 | transcript=AALC636_024560.R34471 | gene=A... 146 5e-35

SCYAPA1_024866.P32618 | transcript=SCYAPA1_024866.R32618 | gene=S... 145 6e-35

CQUJHB012679.P19663 | transcript=CQUJHB012679.R19663 | gene=CQUJH... 145 7e-35

ASTEI02004-PA | transcript=ASTEI02004-RA | gene=ASTEI02004 | orga... 145 1e-34

ADAR2_001930.P21641 | transcript=ADAR2_001930.R21641 | gene=ADAR2... 144 1e-34

AALB20_033171.P66492 | transcript=AALB20_033171.R66492 | gene=AAL... 144 1e-34

AFAF019387-PA | transcript=AFAF019387-RA | gene=AFAF019387 | orga... 144 1e-34

AARA017833-PA | transcript=AARA017833-RA | gene=AARA017833 | orga... 145 1e-34

AARA21_009090.P17039 | transcript=AARA21_009090.R17039 | gene=AAR... 145 1e-34

ACMO_007424.P13503 | transcript=ACMO_007424.R13503 | gene=ACMO_00... 145 1e-34

ADAR2_001930.P21643 | transcript=ADAR2_001930.R21643 | gene=ADAR2... 145 1e-34

ANILCM1_038625.P14110 | transcript=ANILCM1_038625.R14110 | gene=A... 144 1e-34

AARA21_009090.P17044 | transcript=AARA21_009090.R17044 | gene=AAR... 144 1e-34

AARA21_009090.P17042 | transcript=AARA21_009090.R17042 | gene=AAR... 144 1e-34

AARA21_009090.P17038 | transcript=AARA21_009090.R17038 | gene=AAR... 144 1e-34

AARA21_009090.P17037 | transcript=AARA21_009090.R17037 | gene=AAR... 144 1e-34

AARA21_009090.P17033 | transcript=AARA21_009090.R17033 | gene=AAR... 144 1e-34

AALB20_033171.P66528 | transcript=AALB20_033171.R66528 | gene=AAL... 144 1e-34

AALB20_033171.P66526 | transcript=AALB20_033171.R66526 | gene=AAL... 144 1e-34

AALB20_033171.P66524 | transcript=AALB20_033171.R66524 | gene=AAL... 144 1e-34

AALB20_033171.P66522 | transcript=AALB20_033171.R66522 | gene=AAL... 144 1e-34

AALB20_033171.P66521 | transcript=AALB20_033171.R66521 | gene=AAL... 144 1e-34

AALB20_033171.P66520 | transcript=AALB20_033171.R66520 | gene=AAL... 144 1e-34

AALB20_033171.P66518 | transcript=AALB20_033171.R66518 | gene=AAL... 144 1e-34

AALB20_033171.P66515 | transcript=AALB20_033171.R66515 | gene=AAL... 144 1e-34

AALB20_033171.P66514 | transcript=AALB20_033171.R66514 | gene=AAL... 144 1e-34

AALB20_033171.P66509 | transcript=AALB20_033171.R66509 | gene=AAL... 144 1e-34

AALB20_033171.P66508 | transcript=AALB20_033171.R66508 | gene=AAL... 144 1e-34

AALB20_033171.P66507 | transcript=AALB20_033171.R66507 | gene=AAL... 144 1e-34

AALB20_033171.P66505 | transcript=AALB20_033171.R66505 | gene=AAL... 144 1e-34

AALB20_033171.P66503 | transcript=AALB20_033171.R66503 | gene=AAL... 144 1e-34

AALB20_033171.P66502 | transcript=AALB20_033171.R66502 | gene=AAL... 144 1e-34

AALB20_033171.P66500 | transcript=AALB20_033171.R66500 | gene=AAL... 144 1e-34

AALB20_033171.P66499 | transcript=AALB20_033171.R66499 | gene=AAL... 144 1e-34

AALB20_033171.P66497 | transcript=AALB20_033171.R66497 | gene=AAL... 144 1e-34

AALB20_033171.P66496 | transcript=AALB20_033171.R66496 | gene=AAL... 144 1e-34

AALB20_033171.P66495 | transcript=AALB20_033171.R66495 | gene=AAL... 144 1e-34

AALB20_033171.P66494 | transcript=AALB20_033171.R66494 | gene=AAL... 144 1e-34

AALB20_033171.P66491 | transcript=AALB20_033171.R66491 | gene=AAL... 144 1e-34

AALB20_033171.P66490 | transcript=AALB20_033171.R66490 | gene=AAL... 144 1e-34

ACMO_007424.P13499 | transcript=ACMO_007424.R13499 | gene=ACMO_00... 144 2e-34

>AALF007356-PA | transcript=AALF007356-RA | gene=AALF007356 |

organism=Aedes_albopictus_Foshan | gene_product=cadherin [Source:Projected

from Aedes aegypti (AAEL000597) VB Community

Annotation] | transcript_product=cadherin [Source:Projected

from Aedes aegypti (AAEL000597) VB Community Annotation] | location=JXUM01S001733:79044-136584(+)

| protein_length=1569

| sequence_SO=supercontig | SO=protein_coding_gene | is_pseudo=false

Length=1569

Score = 149 bits (375), Expect = 6e-36, Method: Compositional matrix adjust.

Identities = 132/456 (29%), Positives = 218/456 (48%), Gaps = 47/456 (10%)

Query 14 DNSKRNPIAKITSDYQATQKITYRISGVGID--QPPFGIFVVDKNTGDINITAIVDREET 71

D R + D Q I Y ++G GID P F +++ +GDI + +DR++

Sbjct 11 DGQNRKQVTATDGDKDRPQNIVYFLTGQGIDPDNPANSKFDINRTSGDIFVLKPLDRDQP 70

Query 72 ---PSFLITCRALNAQGLDVEKPLILTVKILDINDNPPVFSQQIFMGEIEENSASNSLVM 128

P + T A + G + + V + DINDN P+F Q ++ G + EN + +VM

Sbjct 71 NGRPQWRFTVFAQDEGGEGLVGYADVQVNLKDINDNAPIFPQGVYFGNVTENGTAGMVVM 130

Query 129 ILNATDADEPNH-LNSKIAFKI---VSQEPAGTPMFLLSRNTGEVRTLTNSLDREQASSY 184

+ A D D+PN N+K+ + I V +E G+P+F + +TG ++T LDRE+ Y

Sbjct 131 TMTAVDYDDPNEGTNAKLIYSIEKNVIEEETGSPIFEIEADTGVIKTAVCCLDRERTPDY 190

Query 185 RLVVSGADKDGEGLSTQCECNIKVKDVNDNFPMFRDSQYSARIEENILSS--ELLRFQVT 242

+ V DG GL +I+VKD+ND P F ++ ++E ++ E+ VT

Sbjct 191 SIQVVAM--DGGGLKGTGTASIRVKDINDMPPQFTKDEWFTEVDETDGTNLPEMPILTVT 248

Query 243 DLDEEYTDNWLAVYFFTSGNEGNWFEIQTDPRTNEGI--LKVVKALDYE-QLQS--VKLS 297

DE+ T+ + SG + F + R N+G LK+V+ LDYE QLQS +

Sbjct 249 VHDEDETNKFQYKVIDNSGYGADKFTMV---RNNDGTGSLKIVQPLDYEDQLQSNGFRFR 305

Query 298 IAVKNKAEFHQSVISRYRVQSTPVTIQVINVREGIAFRPASKTFTVQKGISSKKLVDYIL 357

I V +K E + + +Y V + V +++ ++ + +P + ++ + V L

Sbjct 306 IQVNDKGEDNDN--DKYHVAYSWVVVKLRDINDN---KPQFERPNIEVSVYENADVGKTL 360

Query 358 GTYQAIDEDTNKAASNVKYVMGRNDGGYLMIDSKTAEIKFVKNMNRDSTFIVNKTITAEV 417

T++A D D S V Y + R S + +F ++N++ T + + + EV

Sbjct 361 ETFKATDPDQG-GRSKVSYAIDR---------SSDRQRQF--SINQEGTVTIQRQLDREV 408

Query 418 --------LAIDE-YTGKTSTGTVYVRVPDFNDNCP 444

LAID+ KT+T T+ V V D NDN P

Sbjct 409 TPRHQVKILAIDDGIPPKTATATLTVIVQDINDNPP 444

Score = 53.1 bits (126), Expect = 2e-05, Method: Compositional matrix adjust.

Identities = 70/306 (23%), Positives = 125/306 (41%), Gaps = 30/306 (10%)

Query 20 PIAKIT-SDYQATQKITYRI---SGVGIDQPPFGIFVVDKNTGDINITAIVDRE---ETP 72

PI +T D T K Y++ SG G D+ F + + TG + I +D E ++

Sbjct 243 PILTVTVHDEDETNKFQYKVIDNSGYGADK--FTMVRNNDGTGSLKIVQPLDYEDQLQSN 300

Query 73 SFLITCRALNAQGLDVEKPLI------LTVKILDINDNPPVFSQQIFMGEIEENSASNSL 126

F + +N +G D + + VK+ DINDN P F + + EN+

Sbjct 301 GFRFRIQ-VNDKGEDNDNDKYHVAYSWVVVKLRDINDNKPQFERPNIEVSVYENADVGKT 359

Query 127 VMILNATDADEPNHLNSKIAFKIVSQEPAGTPMFLLSRNTGEVRTLTNSLDREQASSYRL 186

+ ATD D+ SK+++ I + + S N T+ LDRE +++

Sbjct 360 LETFKATDPDQGGR--SKVSYAI---DRSSDRQRQFSINQEGTVTIQRQLDREVTPRHQV 414

Query 187 VVSGADKDGEGLSTQCECNIKVKDVNDNFPMFRDSQYSARIEENILSSELLRFQVTDLDE 246

+ D + + V+D+NDN P F Y + E++ +++ TD D+

Sbjct 415 KILAIDDGIPPKTATATLTVIVQDINDNPPKFL-KDYRPVLPEHVPPRKVVEILATDDDD 473

Query 247 EYTDNWLAVYFFTSGNEGNW----FEIQTDPRTNEG----ILKVVKALDYEQLQSVKLSI 298

N F + F+++ D + G I+ +++ D EQ + + I

Sbjct 474 RSKSNGPPFQFRLDPGADDIIRASFKVEQDQKGANGDGMAIVSSLRSFDREQQKEYLIPI 533

Query 299 AVKNKA 304

+K+

Sbjct 534 VIKDHG 539

Score = 43.9 bits (102), Expect = 0.011, Method: Compositional matrix adjust.

Identities = 48/199 (24%), Positives = 73/199 (37%), Gaps = 19/199 (10%)

Query 27 DYQATQKITYRISGVGIDQPPFGIFVVDKNTGDINITAIVDREETPSFLITCRALNAQGL 86

D K++Y I Q F I G + I +DRE TP + A++

Sbjct 369 DQGGRSKVSYAIDRSSDRQRQFSI----NQEGTVTIQRQLDREVTPRHQVKILAIDDGIP 424

Query 87 DVEKPLILTVKILDINDNPPVFSQQIFMGEIEENSASNSLVMILNATDADEPNHLNSKIA 146

LTV + DINDNPP F + + + E+ +V IL D D

Sbjct 425 PKTATATLTVIVQDINDNPPKFLKD-YRPVLPEHVPPRKVVEILATDDDDRSKSNGPPFQ 483

Query 147 FKIVSQEPAGTPMFLLSRNTGEVRTLTN-----------SLDREQASSYRLVVSGADKDG 195

F++ +P + S + + N S DREQ Y + + D

Sbjct 484 FRL---DPGADDIIRASFKVEQDQKGANGDGMAIVSSLRSFDREQQKEYLIPIVIKDHGN 540

Query 196 EGLSTQCECNIKVKDVNDN 214

++ + + DVNDN

Sbjct 541 PAMTGTSTLTVVIGDVNDN 559

**SR2. Peptide Selection and Similarity Assessment**

MHC class II peptide-binding predictions performed using the IEDB tool identified 19 unique predicted peptides within the hDSG3 EC1 domain and 167 within the *A. albopictus* EC1-like domain (Aa_P) (**Suppl. Table S1)**

**SR3. Molecular Dynamics Simulation Details**

**SR3.1 Integration of Structural and Energetic Observations**

Collectively, RMSD, RMSF, and MM/GBSA analyses provided a coherent, internally consistent description of peptide–HLA interaction behavior. Peptides prioritized through immunogenetic and sequence-based filtering demonstrate stable structural engagement with HLA molecules and energetically favorable interaction profiles within the limits of silico modeling. These supplementary results support the computational plausibility of the selected vector-derived candidates as potential HLA binders. However, they are presented as descriptive and hypothesis-generating observations rather than definitive evidence of functional immune recognition or pathogenic relevance.


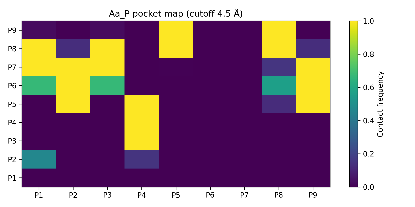

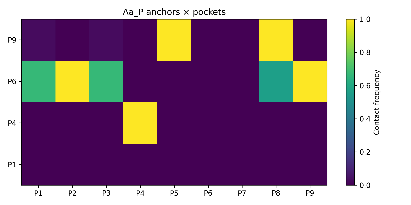


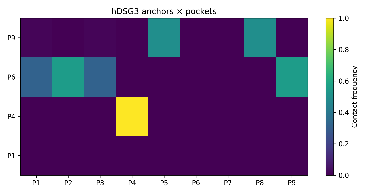

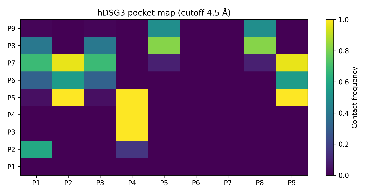


**Figure S1.** Residue-level contact map of the vector-derived peptide bound to HLA-DRB1*04:02.

Residue–residue contact maps were generated from the equilibrated portion of the molecular dynamics trajectory using a distance-based cutoff between peptide and HLA atoms. Heatmap color intensity indicates the frequency of contacts observed across analyzed frames, with higher values reflecting more persistent interactions. Panels depict overall peptide–HLA contacts as well as contacts mapped onto canonical MHC class II anchor positions (P1–P9). These maps provide a descriptive, spatially resolved representation of peptide engagement within the HLA binding groove and are intended to complement RMSD, RMSF, and MM/GBSA analyses rather than to infer functional or pathogenic significance.


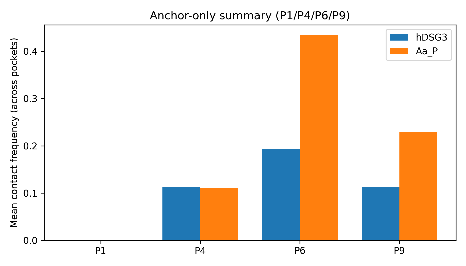


**Figure S2.** Anchor-pocket contact frequency summary for peptide–HLA interactions.

Bar plots summarize the mean contact frequencies between peptide anchor positions (P1, P4, P6, and P9) and the corresponding HLA binding groove pockets, calculated from equilibrated molecular dynamics trajectories. Contact frequencies represent the fraction of simulation frames in which at least one peptide–HLA residue pair at the indicated anchor position satisfied the contact distance criterion. Blue bars correspond to the hDSG3-derived peptide, and orange bars correspond to the vector-derived peptide (Aa_P). These values are presented for descriptive comparison of binding mode characteristics and do not imply differences in immunogenicity or pathogenic relevance.


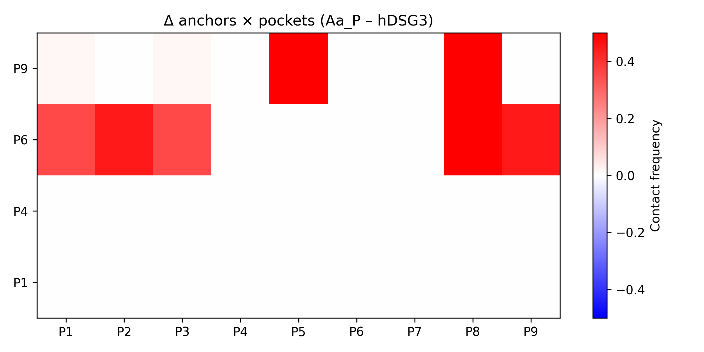

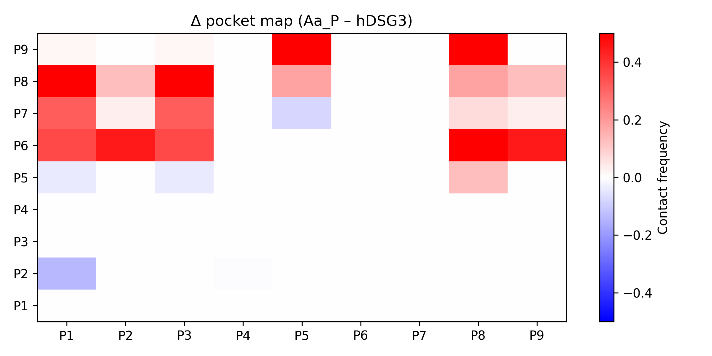


**Figure S3.** Residue-level contact patterns of vector-derived and hDSG3-derived peptides bound to HLA-DRB1*04:02.

Residue-level contact maps were generated from the equilibrated portions of the molecular dynamics trajectories to visualize persistent interactions between peptides and the HLA-DRB1*04:02 binding groove. Contacts were defined using a distance-based criterion between peptide and HLA residues, and contact frequencies were calculated across analyzed frames. Color intensity reflects the persistence of contact over time, with warmer colors indicating higher contact frequency. The upper panels depict contact maps highlighting interactions between peptide residues and canonical HLA binding pockets. The lower panel summarizes mean contact frequencies at MHC class II anchor positions (**P1, P4, P6,** and **P9**), calculated from equilibrated trajectories. Comparison between the vector-derived peptide and the hDSG3-derived peptide shows broadly similar anchor engagement patterns, with modest differences in contact persistence. These analyses are presented for comparative and descriptive purposes and are not interpreted as indicators of differential immunogenicity or pathogenic potential.

**SR4. RMSD and RMSF Analyses**

Figures related to this section are given in the main text.

**SR5. Comparative Binding Free Energy Estimates**

Binding free energy estimates obtained using the MM/GBSA approach revealed consistent interaction energetics across the analyzed peptide–HLA complexes. While absolute ΔGbinding values varied between peptides, the relative ranking of binding energies was stable across analyzed frames. Per-residue energy decomposition analyses indicated that peptide positions corresponding to predicted anchor residues contributed disproportionately to overall binding stabilization. These energetic contributions were primarily driven by van der Waals and electrostatic interactions within the HLA binding groove. Solvation energy components partially offset favorable interaction energies, as expected for peptide–MHC class II complexes. These binding free energy estimates were used exclusively for relative comparison between peptide systems. Given the inherent limitations of MM/GBSA methods, including sensitivity to force field parameters and sampling depth, the reported values were not interpreted as absolute binding affinities.


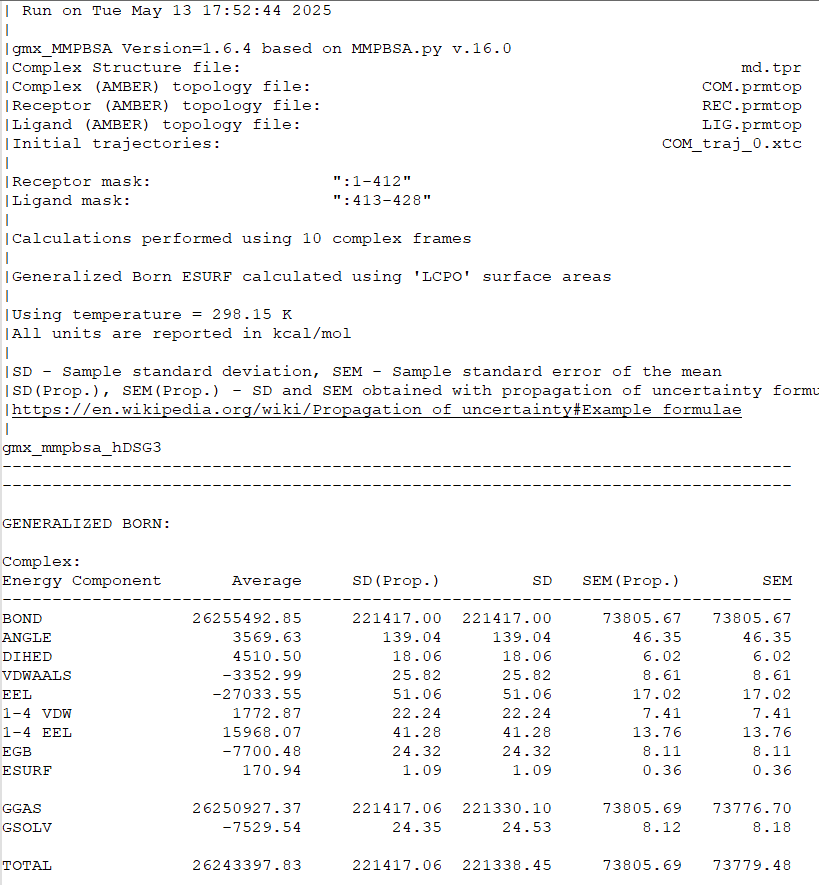


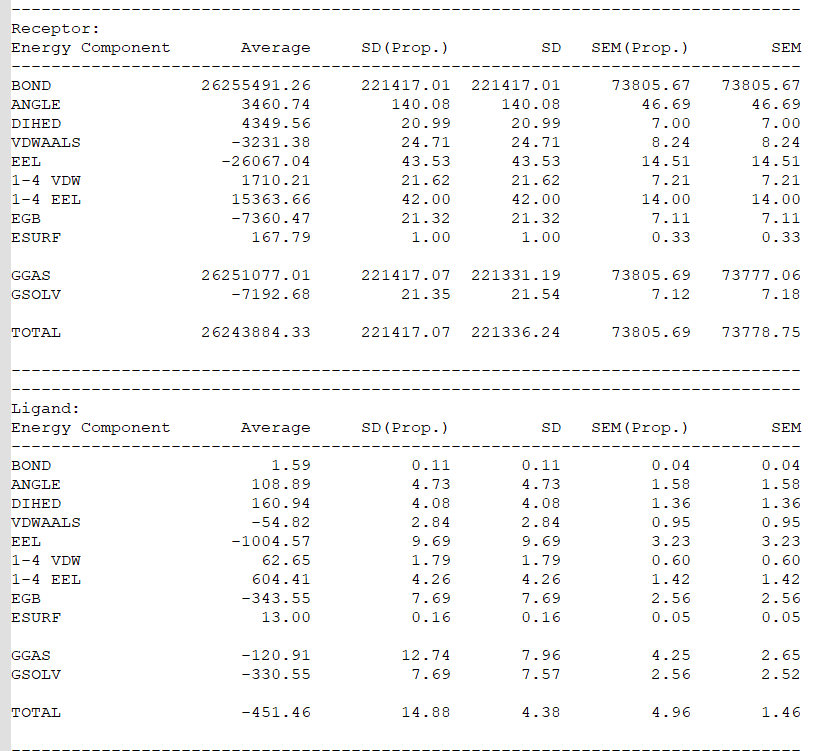


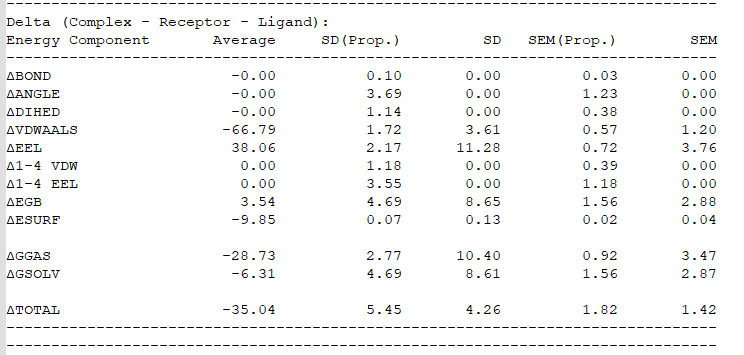


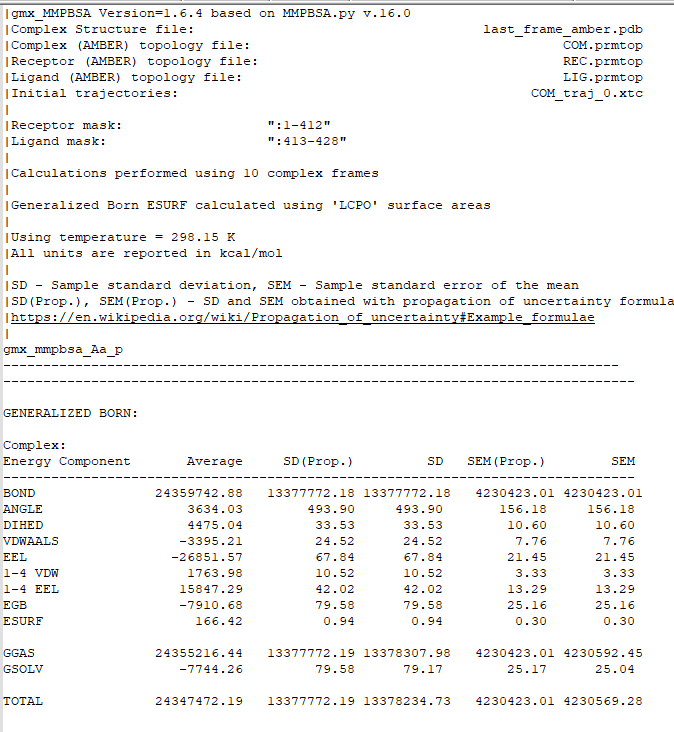


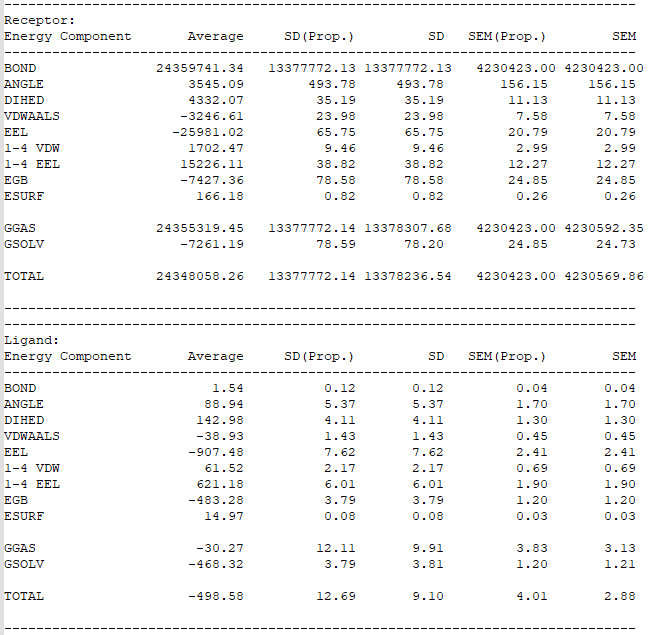


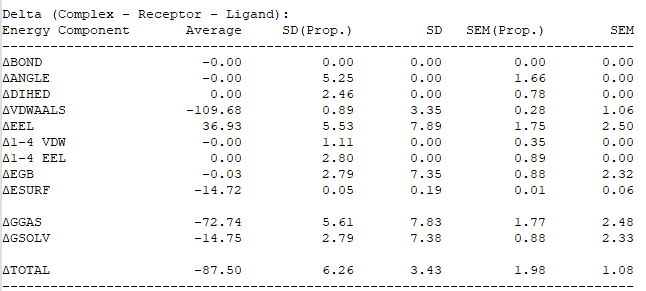

Supplement: Supplementary file 1 [file DataSheet1.docx]
